# 3D Analysis of the Synaptic Organization in the Entorhinal Cortex in Alzheimer’s Disease

**DOI:** 10.1523/ENEURO.0504-20.2021

**Published:** 2021-06-18

**Authors:** M. Domínguez-Álvaro, M. Montero-Crespo, L. Blazquez-Llorca, S. Plaza-Alonso, N. Cano-Astorga, J. DeFelipe, L. Alonso-Nanclares

**Affiliations:** 1 Laboratorio Cajal de Circuitos Corticales, Centro de Tecnología Biomédica, Universidad Politécnica de Madrid, Madrid 28223, Spain; 2 Instituto Cajal, Consejo Superior de Investigaciones Científicas (CSIC), Madrid 28002, Spain; 3 Centro de Investigación Biomédica en Red sobre Enfermedades Neurodegenerativas (CIBERNED), Instituto de Salud Carlos III 28031, Madrid, Spain; 4 Sección Departamental de Anatomía y Embriología (Veterinaria), Facultad de Veterinaria, Universidad Complutense de Madrid, Madrid 28040, Spain

**Keywords:** dementia, electron microscopy, FIB/SEM, medial temporal lobe, neuropil, postsynaptic targets

## Abstract

The entorhinal cortex (EC) is especially vulnerable in the early stages of Alzheimer’s disease (AD). In particular, cognitive deficits have been linked to alterations in the upper layers of EC. In the present report, we examined Layers II and III from eight human brain autopsies (four subjects with no recorded neurologic alterations and four AD cases). We used stereological methods to assess cortical atrophy of the EC and possible changes in the volume occupied by different cortical elements (neuronal and glial cell bodies; blood vessels; and neuropil). We performed 3D ultrastructural analyses of synapses using focused ion beam/scanning electron microscopy (FIB/SEM) to examine possible alterations related to AD. At the light microscope level, we found a significantly lower volume fraction occupied by neuronal bodies in Layer III and a higher volume fraction occupied by glial cell bodies in Layer II in AD cases. At the ultrastructural level, we observed that (1) there was a significantly lower synaptic density in both layers in AD cases; (2) synapses were larger and more complex in Layer II in AD cases; and (3) there was a greater proportion of small and simple synapses in Layer III in AD cases than in control individuals. These structural differences may play a role in the anatomic basis for the impairment of cognitive functions in AD.

## Significance Statement

Analysis of the synaptic characteristics provides critical data on synaptic organization. Using 3D electron microscopy (EM), the present study shows the synaptic organization of the neuropil of the human entorhinal cortex (EC) at the ultrastructural level. The EC is especially vulnerable in the early stages of Alzheimer’s disease (AD). Our present results show structural differences that may contribute as anatomic basis for the impairment of cognitive functions in AD. Thus, these results may help to understand the relationship between alterations of the synaptic circuits and the cognitive deterioration in AD.

## Introduction

Alzheimer’s disease (AD) is a progressive neurodegenerative disease which is considered to be the main cause of dementia. The brain of AD cases shows brain atrophy and, at the neuropathological level, the most characteristic findings include the presence of extracellular amyloid-β (Aβ) plaques and intracellular neurofibrillary tangles (NFTs) of filamentous aggregates of hyperphosphorylated tau protein (Alzheimer’s Association, 2020). Aβ plaques and NFTs are mostly found in the cerebral cortex, where both their numbers and the proportion of the cortex affected by them increase progressively as the disease advances (Braak and Braak, 1991; Dickson, 1997; Thal et al., 2002). Studies focusing on AD progression have shown that entorhinal cortex (EC) is one of the first brain regions affected by the presence of altered tau protein (Braak and Braak, 1991).

The EC has been shown to be essential for memory functions and spatial navigation (for review, see Schultz et al., 2015), and alterations in its upper layers have been related to cognitive deficits in AD individuals (Van Hoesen et al., 1991; Gómez-Isla et al., 1996). EC is considered to be an interface between the hippocampal formation and a large variety of association and limbic cortices (Solodkin and Van Hoesen, 1996; Lavenex and Amaral, 2000). In particular, the EC is the origin of the perforant pathway (from Layers II and III), which provides the largest input source to the hippocampal formation, targeting the ammonic fields (CA) CA1, CA2, and CA3, as well as the dentate gyrus (DG) and subiculum. Specifically, Layer II neurons project to CA1 via the trisynaptic circuit, passing through the DG and CA3, while Layer III neurons project directly to CA1 in the monosynaptic pathway (Insausti and Amaral, 2012; Kondo et al., 2009). It has been proposed that the trisynaptic pathway is more susceptible to premature degeneration (for review, see Van Hoesen et al., 2006; Llorens-Martín et al., 2014).

The presence of pathologic forms of Aβ and tau proteins in the cerebral cortex of AD cases has been related to neuronal loss, synapse alterations and dendritic spine degeneration (for review, see Forner et al., 2017; Chen et al., 2019). The loss and dysfunction of synapses have been proposed as the major structural correlates of the cognitive decline associated with AD (Coleman et al., 2004; Dickson et al., 1995; Selkoe, 2002; Sze et al., 1997). It has been proposed that synaptic loss affects the subcortical regions and the EC first and then progresses to other cortical regions (Braak and Braak, 1991; Braak and Del Tredici, 2012, 2020). Therefore, deciphering the changes that affect the normal function of synapses may contribute to better understanding of the pathologic mechanisms of AD.

In the present study, we performed 3D ultrastructural analysis of the EC using focused ion beam/scanning electron microscopy (FIB/SEM) and specific software that allows the segmentation of synapses in a reconstructed 3D volume (Morales et al., 2011). This technology greatly facilitates the analysis of possible alterations at the synaptic level in AD, as previously shown in the human transentorhinal cortex and the CA1 hippocampal region (Domínguez-Álvaro et al., 2019; Montero-Crespo et al., 2021). Our goal was to investigate the possible synaptic changes occurring in Layers II and III of the EC related to AD, not only with regard to numbers, spatial distribution and types of synapses, but also regarding the morphologic characteristics of each synapse (shape and size), as well as possible changes in their postsynaptic targets. For this purpose, we performed an analysis of the neuropil from Layers II and III of the EC, from eight human brain autopsies (four subjects with no recorded neurologic alterations and four AD cases) with short postmortem delays (<3.5 h). Since the presence of Aβ plaques is related to a virtual lack of synapses in their vicinity (Blazquez-Llorca et al., 2013), we focused on the neuropil that was free of plaques. We also used stereological methods to assess cortical atrophy of the EC, and possible changes in the volume occupied by different cortical elements (neuronal and glial cell bodies, blood vessels and neuropil) at the light microscope level.

## Materials and Methods

### Tissue preparation

Human brain tissue was obtained from eight autopsies (four male and four female subjects) with short postmortem delays (<3.5 h; supplied by Instituto de Neuropatología del IDIBELL-Hospital Universitario de Bellvitge, Barcelona, Spain; Unidad Asociada Neuromax, Laboratorio de Neuroanatomía Humana, Facultad de Medicina, Universidad de Castilla-La Mancha, Albacete and the Laboratorio Cajal de Circuitos Corticales, Universidad Politécnica de Madrid-Consejo Superior de Investigaciones Científicas). The sampling procedure was approved by the Institutional Ethical Committees of each of the institutions involved. Tissue from some of these human brains has been used in previous studies (Domínguez-Álvaro et al., 2018, 2019, 2021; Montero-Crespo et al., 2020, 2021; Cano-Astorga et al., 2021).

Briefly, tissue samples were obtained from four control cases (non-demented subjects with no recorded neurologic or psychiatric alterations) and four AD cases according to the neuropathological criteria provided by the above-mentioned centers (Table 1; Mirra et al., 1991).

**Table 1:** Clinical and neuropathological information

Case	Sex	Age (years)	Cause of death	Postmortem delay (h)	Braak stage	CERAD stage	Neuropsychological diagnosis
AB1	Male	45	Lung cancer	<1	0	0	No recorded neurologic alterations
AB3	Male	53	Bladder carcinoma	3.5	0	0	No recorded neurologic alterations
IF10	Male	66	Bronchopneumonia and cardiac failure	2	0	0	No recorded neurologic alterations
M16	Male	40	Traffic accident	3	0	0	No recorded neurologic alterations
IF1	Female	80	-	2	IV	B	No evidence of cognitive impairment and dementia
VK11	Female	87	Respiratory inflammation	1.5	III−IV	A	Dementia
VK16	Female	88	-	2.5	VI	C	Dementia
VK22	Female	86	-	2	V	C	Dementia

Braak stages (Braak and Braak, 1991): III, NFTs in EC and closely related areas; III−IV, NFTs abundant in amygdala and hippocampus; extending slightly into association cortex; V−VI, NFTs widely distributed throughout the neocortex and ultimately involving primary motor and sensory areas. CERAD stages (Mirra et al., 1991): A, low density of neuritic plaques; B, intermediate density of neuritic plaques; C, high density of neuritic plaques. -, not available.

**Table 2:** Accumulated data obtained from the ultrastructural analysis of the neuropil from Layers II and III of the EC

EC layer	Group	No. AS	No. SS	No. all synapses	% AS (mean ± SD)	% SS (mean ± SD)	CF volume (μm^3^)	No. AS/μm^3^ (mean ± SD)	No. SS/μm^3^ (mean ± SD)	No. all synapses/μm^3^ (mean ± SD)	Distance to nearest neighbor (nm; mean ± SD)
II	Control	1553	137	1690	91.90 ± 2.62	8.10 ± 2.62	4221 (5445)	0.37 ± 0.05 (0.38 ± 0.06)	0.03 ± 0.01 (0.03 ± 0.01)	0.40 ± 0.03 (0.41 ± 0.06)	840 ± 35 (811 ± 33)
	Alzheimer	954	90	1044	90.53 ± 4.27	9.47 ± 4.27	4409 (5742)	0.21 ± 0.09 (0.23 ± 0.09)	0.02 ± 0.01 (0.02 ± 0.01)	0.24 ± 0.09 (0.25 ± 0.09)	938 ± 113 (906 ± 109)
III	Control	1747	130	1877	92.84 ± 1.97	7.16 ± 1.978	4371 (5466)	0.40 ± 0.09 (0.42 ± 0.09)	0.03 ± 0.01 (0.03 ± 0.01)	0.43 ± 0.01 (0.45 ± 0.09)	852 ± 42 (823 ± 41)
	Alzheimer	973	92	1065	92.12 ± 5.70	7.88 ± 5.70	4597 (5653)	0.22 ± 0.06 (0.23 ± 0.08)	0.02 ± 0.02 (0.02 ± 0.02)	0.24 ± 0.08 (0.25 ± 0.09)	1004 ± 214 (970 ± 206)

All volume data are corrected for shrinkage factor. Data in parentheses are not corrected with the shrinkage and the fixation artifact factors. The data for individual cases are shown in Extended Data Table 2-1.

10.1523/ENEURO.0504-20.2021.tab2-1 Extended Data Table 2-1 Data from the ultrastructural analysis of neuropil from Layers II and III of the EC for individual cases. All volume and distance data are corrected for shrinkage. All, includes AS+SS synapses; No., number. Download Table 2-1, DOCX file.

**Figure 1.**
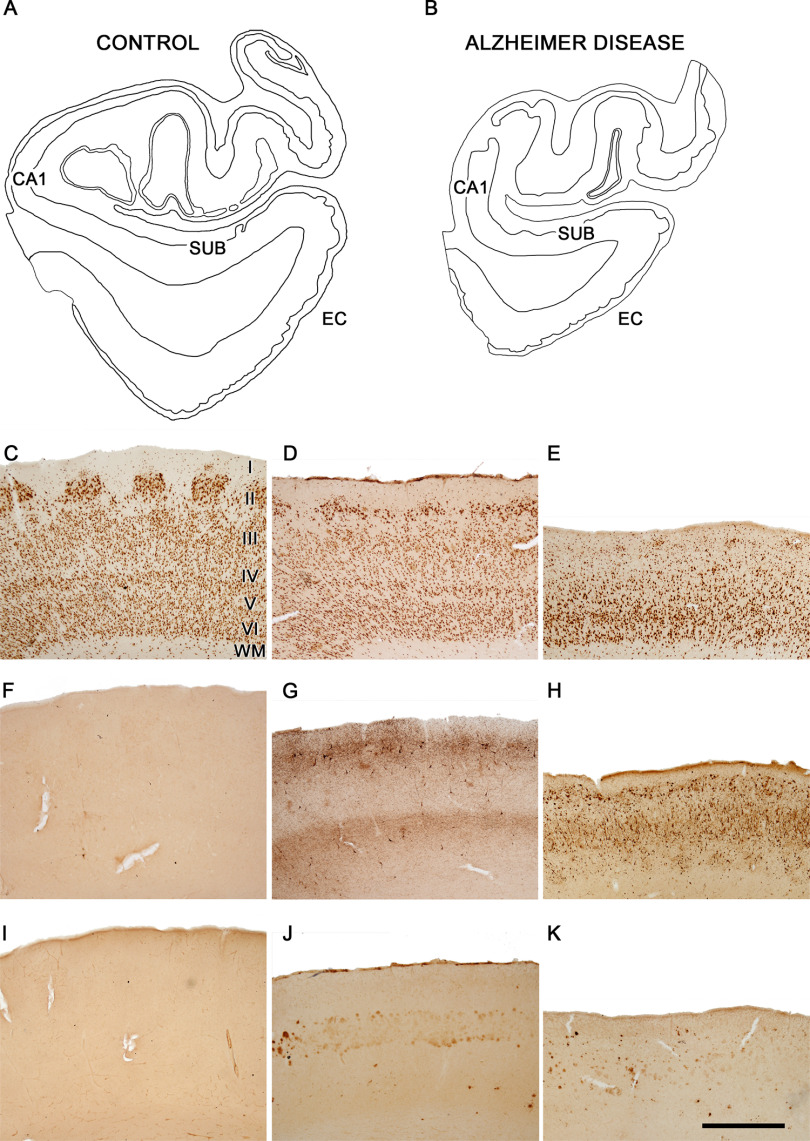
EC of control and AD cases. Plots of the medial structures including the hippocampal formation and EC from control (***A***) and AD (***B***) cases to illustrate the atrophy that occurs in AD. Plots are based on Nissl-stained coronal sections from cases AB3 and IF1. Microphotographs showing the EC in a control case AB3 (***C***, ***F***, ***I***) and in AD cases IF1 (***D***, ***G***, ***J***) and VK16 (***E***, ***H***, ***K***). Layers are indicated to the right of panel ***C***. Sections are immunostained with antibodies anti-NeuN (***C–E***), anti-PHF-Tau-AT8 (***F–H***), and anti-Aβ (***I–K***). Note the intense labeling of PHF-Tau-AT8-positive neurons (***G***, ***H***) and Aβ-positive plaques (***J***, ***K***) in AD cases, particularly in case VK16, and the lack of labeling in the control case. CA1, cornu ammonis field 1; SUB, subiculum; WM, white matter. Scale bar in ***K***: 4 mm (***A***, ***B***) and 1 mm (***C−K***).

Upon removal, the brain tissue was fixed in cold 4% paraformaldehyde (Sigma-Aldrich) in 0.1 m sodium phosphate buffer (PB; Panreac, 131965), pH 7.4, for 24–48 h. After fixation, the tissue was washed in PB and coronally sectioned (150 μm thick) in a vibratome (Vibratome Sectioning System, VT1200S Vibratome, Leica Biosystems). Sections containing EC were selected for Nissl staining, immunohistochemistry, and EM processing (Fig. 1).

Coronal sections of the human EC at medial level were used for the present study (for review, see Insausti et al., 2017). The delimitation of the EC was established by combining the Nissl and anti-NeuN markers (Fig. 1). The main cytoarchitectural characteristic of the EC that allows it to be recognized is the presence of large islands of modified pyramidal neurons and stellate cells in Layer II (Braak and Braak, 1992; Insausti and Amaral, 2012; Kobro‐Flatmoen and Witter, 2019). Sections containing EC were selected for Nissl staining, immunohistochemistry and EM processing (Fig. 1).

### Immunohistochemistry

The selected sections were first rinsed in 0.1 m PB, pretreated in 2% H_2_O_2_ for 30 min to remove endogenous peroxidase activity, and then incubated for 1 h at room temperature in a solution of 3% normal horse serum (for monoclonal antibodies; Vector Laboratories Inc.) and 0.25% Triton X-100 (Merck). The sections were then incubated for 48 h at 4°C in the same solution with mouse anti-NeuN (1:2000; Chemicon; MAB377) and anti-human PHF_-Tau_ antibody clone AT8 (1:2000, MN1020, Thermo Scientific); for the sake of clarity, we will refer to this as anti-PHF_-Tau-AT8_. The sections selected for anti-Aβ were first treated with 88% formic acid (Sigma-Aldrich, #251364) to ensure specific plaque immunostaining, and were then incubated in a solution containing mouse antibody anti-Aβ (clone 6F/3D; 1:50, Dako M0872). The sections were then processed with a secondary biotinylated horse anti-mouse IgG antibody (1:200, Vector Laboratories), and then incubated for 1 h in an avidin-biotin peroxidase complex (Vectastain ABC Elite PK6100, Vector) and, finally, with the chromogen 3,3′-diaminobenzidine tetrahydrochloride (DAB; Sigma-Aldrich). Finally, the sections were dehydrated, cleared with xylene, and cover-slipped.

### Tissue processing for EM

EC sections were postfixed for 24 h in a solution containing 2% paraformaldehyde, 2.5% glutaraldehyde (TAAB, G002) and 0.003% CaCl_2_ (Sigma, C-2661-500G) in sodium cacodylate (Sigma, C0250-500G) buffer (0.1 m). These sections were washed in sodium cacodylate buffer (0.1 m) and treated with 1% OsO_4_ (Sigma, O5500), 0.1% potassium ferrocyanide (Probus, 23345) and 0.003% CaCl_2_ in sodium cacodylate buffer (0.1 m) for 1 h at room temperature. After washing in PB, the sections were stained with 2% uranyl acetate (EMS, 8473), and then dehydrated and flat-embedded in Araldite (TAAB, E021) for 48 h at 60°C (DeFelipe and Fairén, 1993). Embedded sections were glued onto a blank Araldite block and trimmed. Semithin sections (1–2 μm thick) were obtained from the surface of the block and stained with 1% toluidine blue (Merck, 115930) in 1% sodium borate (Panreac, 141644).

**Figure 2.**
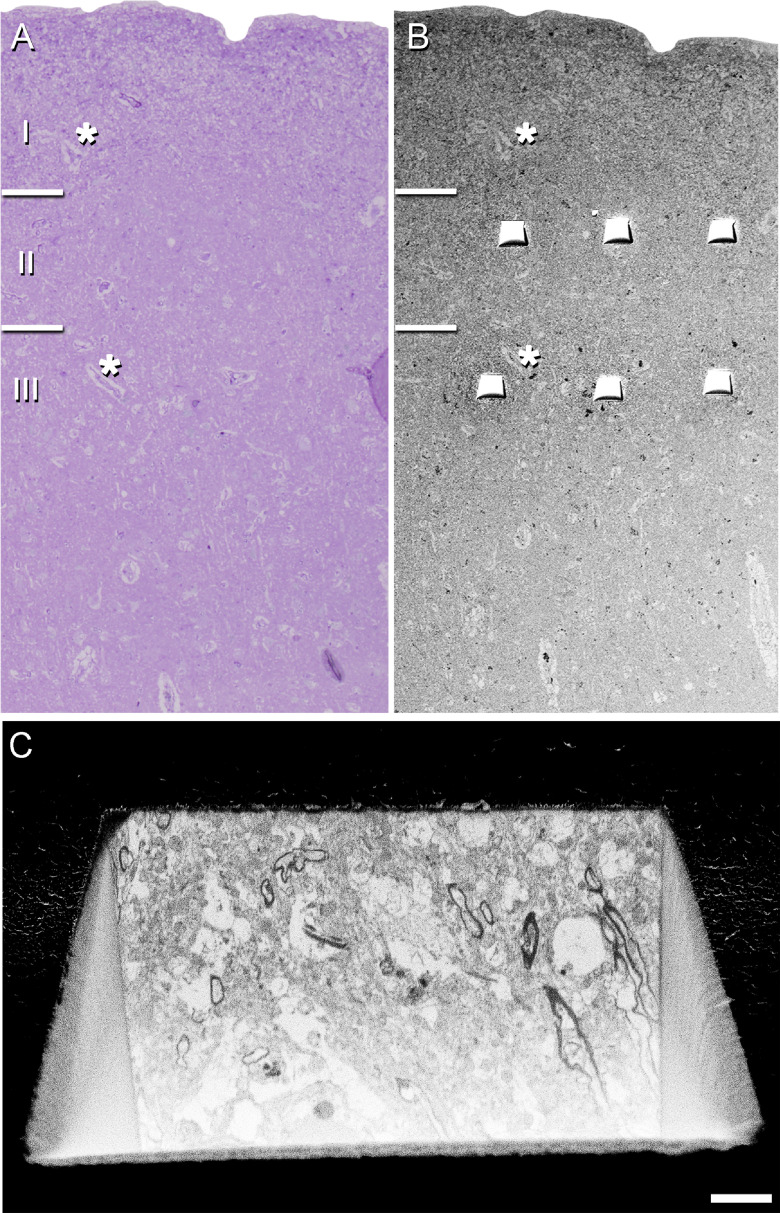
Correlative light/EM of Layers II and III of the EC. Delimitation of layers is based on the staining pattern of 1-μm-thick semithin section, stained with toluidine blue (***A***), which is adjacent to the block for FIB/SEM imaging (***B***). ***B***, SEM image illustrating the block surface with trenches made in the neuropil (three per layer). Asterisks in ***A***, ***B*** point to the same blood vessels, showing that the exact location of the region of interest was accurately determined. ***C***, SEM image showing the front of a trench made to expose the tissue and to acquire the FIB/SEM stack of images at the final magnification. Scale bar in ***C***: 70 μm (***A***), 85 μm (***B***), and 3 μm (***C***).

The last semithin section (which corresponds to the section immediately adjacent to the block surface) was examined under light microscope and photographed to accurately locate the neuropil regions to be examined (Fig. 2). The blocks containing the embedded tissue were then glued onto a sample stub using conductive adhesive tabs (EMS 77825-09). All the surfaces of the block, except for the one to be studied (the top surface), were covered with silver paint (EMS 12630) to prevent charging artifacts. The stubs with the mounted blocks were then placed into a sputter coater (Emitech K575X, Quorum Emitech) and the top surface was coated with a 10- to 20-nm-thick layer of gold/palladium to facilitate charge dissipation.

### Cortical thickness estimation

In order to estimate the atrophy of the EC, we measured the cortical thickness in three to five toluidine blue-stained semithin sections from each of the cases, obtained in the coronal plane of the cortex and containing the entire cortex, tracing a perpendicular line from the pial surface to the white matter. Measurements of the distance between the pial surface and the boundary with the white matter were performed using Fiji program (ImageJ 1.51; National Institutes of Health; http://imagej.nih.gov/ij/). To average data, three measurements were made per section.

### Volume fraction estimation of cortical elements

Three to five semithin sections (1–2 μm thick) stained with 1% toluidine blue were used to estimate the respective volume fractions (Vv) occupied by blood vessels, cell bodies (glia and neurons) and neuropil. This estimation was performed applying the Cavalieri principle (Gundersen et al., 1988) by point counting using the integrated Stereo Investigator stereological package (version 8.0, MicroBrightField Inc.) attached to an Olympus light microscope (Olympus) at 40× magnification. A grid, whose points covered an area of 400 μm^2^, was overlaid over each semithin section to determine the Vv occupied by the different elements: neurons, glia, blood vessels, and neuropil.

### 3D EM

The Araldite block containing the tissue was used to obtain images stacks from the EC (Fig. 2) using a dual beam microscope (FIB/SEM; Crossbeam 540 EM, Carl Zeiss NTS GmbH).

The dual beam microscope combines a high-resolution field-emission SEM column with a focused gallium ion beam (FIB), which permits removal of thin layers of material from the sample surface on a nanometer scale. As soon as one layer of material (20 nm thick) is removed by the FIB, the exposed surface of the sample is imaged by the SEM using a backscattered electron detector. The sequential automated use of FIB milling and SEM imaging allowed us to obtain long series of photographs of a 3D sample of selected regions (Merchán-Pérez et al., 2009). FIB/SEM images from the neuropil were obtained avoiding the neuronal and glial somata, blood vessels and also avoiding Aβ plaques to eliminate the effect of alterations of synapses in the vicinity of Aβ-plaques, which has been described previously (Blazquez-Llorca et al., 2013).

In total, 24 stacks of images of the neuropil from Layers II and III of the EC from AD cases were obtained (three stacks for each of the 4 cases, with a total volume studied of 9006 μm^3^; Table 2). The data obtained from these stacks of images were compared with the data obtained in the same regions of four control cases (Domínguez-Álvaro et al., 2021).

**Table 3:** Area (mean ± SEM, in nm^2^) and perimeter (mean ± SEM, in nm) of the SAS in Layers II and III of the EC

EC layer	Group	Type of synapse (no. of synapses)	Area of SAS	Perimeter of SAS
II	Control	AS (1552)	110,311 ± 3229 (102,865)	1631 ± 47 (1575)
		SS (139)	65,997 ± 6041 (61,543)	1404 ± 73 (1356)
	Alzheimer	AS (952)	136,166 ± 10,773 (126,976)	1819 ± 52 (1757)
		SS (90)	59,232 ± 6589 (55,234)	1239 ± 72 (1196)
III	Control	AS (1746)	124,183 ± 3265 (115,801)	1826 ± 67 (1763)
		SS (129)	67,445 ± 3243 (62,893)	1399 ± 75 (1351)
	Alzheimer	AS (973)	122,727 ± 10,453 (114,444)	1720 ± 116 (1661)
		SS (94)	57,271 ± 14,132 (56,082)	1343 ± 272 (1348)

Data on area and perimeter are corrected for shrinkage factor (data in parentheses are not corrected with the shrinkage and the fixation artifact factors). The data for individual cases are shown in Extended Data Table 3-1.

10.1523/ENEURO.0504-20.2021.tab3-1 Extended Data Table 3-1 Area (nm^2^) and perimeter (nm) of the SAS in Layers II and III of the EC for individual cases. All data are corrected for shrinkage factor. Download Table 3-1, DOCX file.

### Synaptic 3D analysis

**Figure 3.**
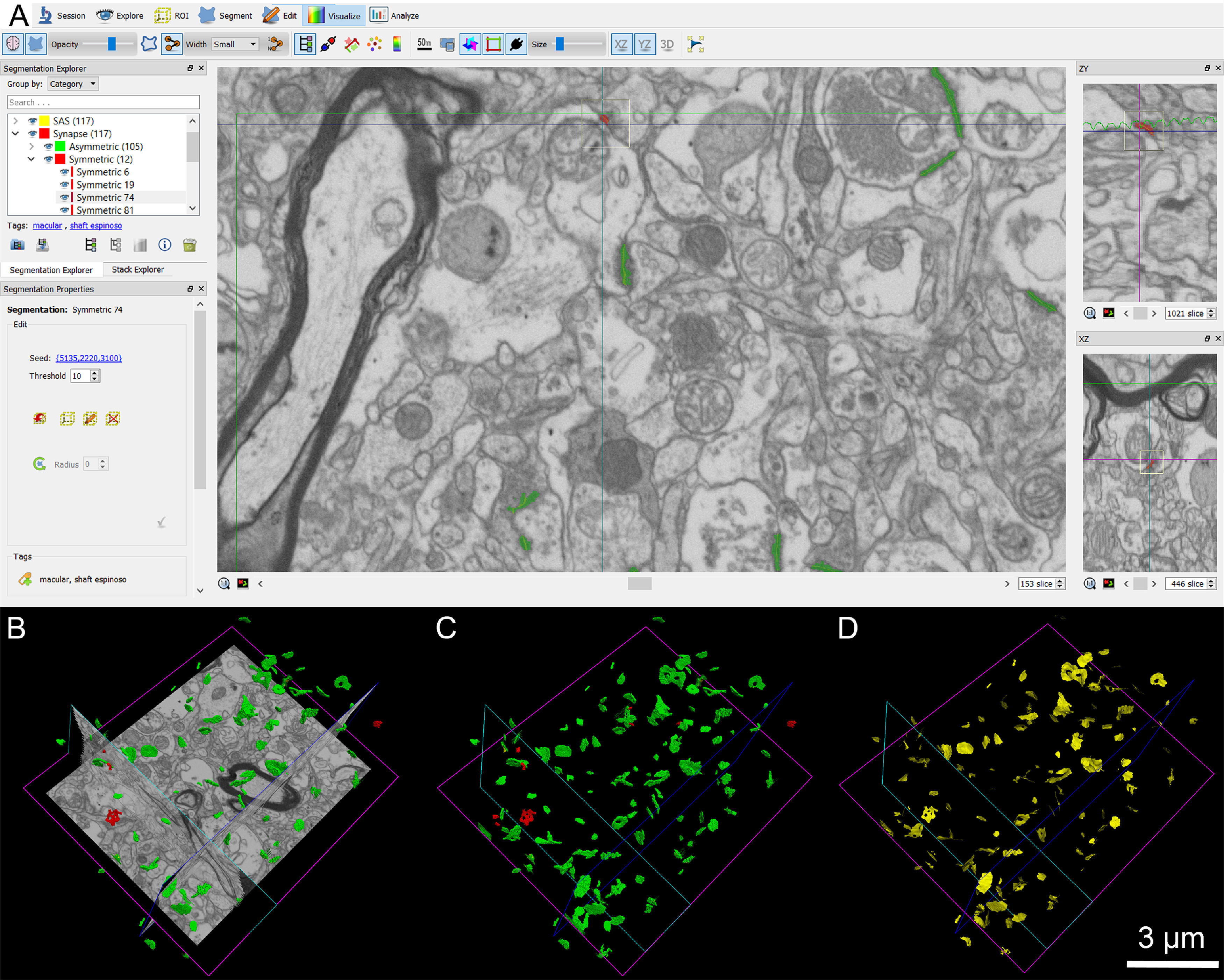
Screenshot of the EspINA software user interface. ***A***, In the main window, the sections are viewed through the *xy* plane (as obtained by FIB/SEM microscopy). The other two orthogonal planes, *yz* and *xz*, are also shown in adjacent windows on the right. ***B***, The 3D windows show the three orthogonal planes and the 3D reconstruction of AS (green) and SS (red) segmented synapses, the reconstructed synapses (***C***), and the computed SAS for each reconstructed synapse (in yellow; ***D***).

**Figure 4.**
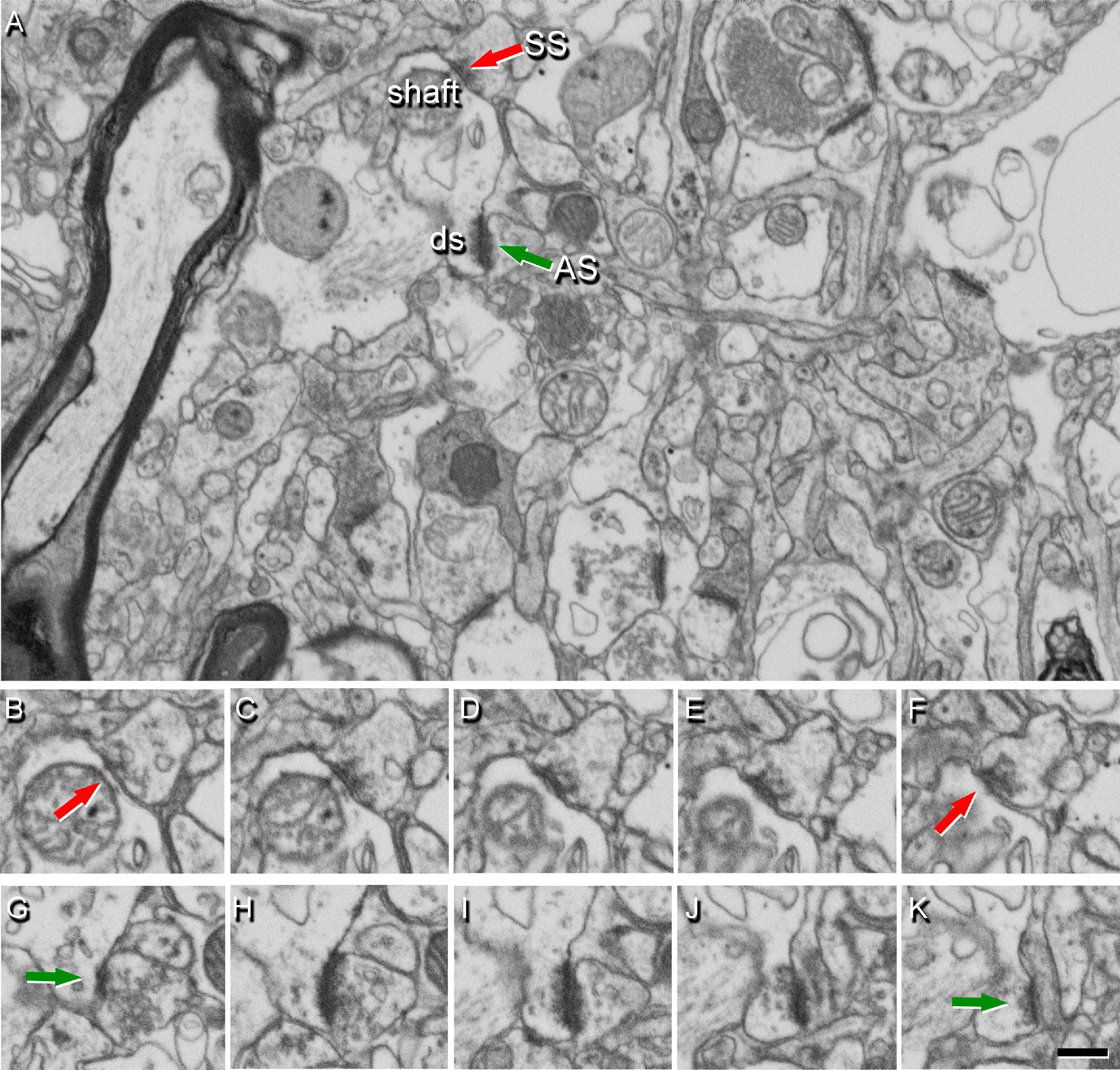
Identification of synapses in serial EM images obtained by FIB/SEM. ***A***, Image from Layer II showing the neuropil from an AD case, with two synapses indicated (arrows) as examples of SS (red) on a dendritic shaft, and AS (green) on a dendritic spine head (ds). Synapse classification was based on examination of the full sequence of serial images; the SS can be visualized in ***B−F***, and the AS in ***G−K***. Scale bar in ***K***: 460 nm (***A***) and 360 nm (***B−K***).

FIB/SEM stacks of images were analyzed using EspINA software (EspINA Interactive Neuron Analyzer, 2.1.9; https://cajalbbp.es/espina/), which allows the segmentation of synapses in the reconstructed 3D volume (Morales et al., 2011; Fig. 3). Since the EspINA software allows navigation through the stack of images (Figs. 3, 4; Movies 1, 2), it was possible to unambiguously identify every synapse as asymmetric synapses (AS) or symmetric synapses (SS), based on the thickness of the PSD (Fig. 4). There is a consensus for classifying cortical synapses into AS (or Type I) and SS (or Type II). The main characteristic distinguishing these synapses is the prominent or thin postsynaptic density, respectively: synapses with prominent PSDs are classified as AS, while thin PSDs are classified as SS (Gray, 1959; Peters and Palay, 1996).

**Figure 5.**
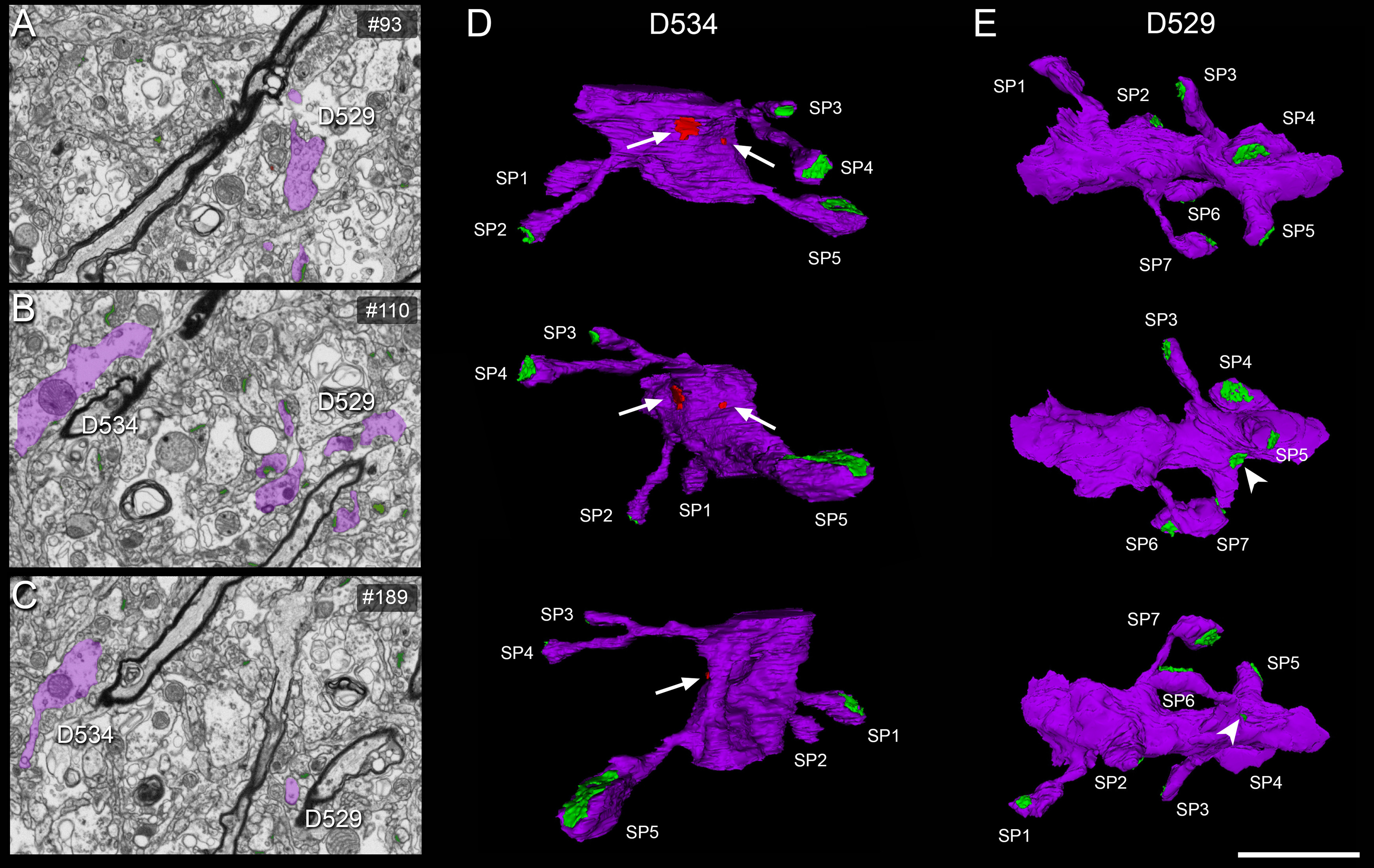
3D reconstruction of dendritic segments from FIB/SEM serial images. ***A–C***, Serial images showing two dendritic segments (D529, D534) partially reconstructed (in purple). 3D reconstructions of these dendritic segments are displayed in panels ***D***, ***E***. ***D***, 3D reconstructed dendritic segment D534 in three views after rotation about the major dendritic axis. Five dendritic spines (SP 1–5) are shown, establishing AS (green), and two SS (arrows) on the shaft (red) are also visible. ***E***, 3D reconstructed dendritic segment D534 in three views after rotation about the major dendritic axis. Seven dendritic spines (SP 1–7) establishing AS (green) are illustrated. Note that the synapse on SP4 can be identified as perforated in the middle panel (***E***). The arrowhead indicates an AS on a spine neck. Scale bar in ***E***: 3 μm (***A–C***) and 2 μm (***D***, ***E***).

Movie 1. EspINA software user interface. FIB/SEM sections are viewed through the *xyz* planes. AS (green) are shown, established on dendritic spines originating from a dendritic shaft (purple), shown as D529 in Figure 5. 10.1523/ENEURO.0504-20.2021.video.1

Movie 2. EspINA software 3D viewer. A stack of images is fully represented in the three orthogonal planes (*x*, *y*, and *z*). The video shows the 3D reconstruction of a single dendritic segment (purple; D529 shown in Fig. 5) and the 3D reconstruction of all AS (green) and SS (red) established in this 3D reconstructed fragment. 10.1523/ENEURO.0504-20.2021.video.2

EspINA also allowed the application of an unbiased 3D counting frame (CF) to obtain the synaptic density per volume (for details, see Merchán-Pérez et al., 2009). The synaptic density values were obtained by dividing the total number of synapses by the total volume of the CF. Geometrical characteristics, such as size, and spatial distribution features (centroids) of each reconstructed synapse were also calculated by EspINA.

EspINA software extracts the synaptic apposition surface (SAS) and provides its morphologic measurements (Fig. 3). Since the presynaptic and postsynaptic densities are located face to face, their surface areas are comparable (for details, see Morales et al., 2013). Since the SAS comprises both the active zone and the PSD, it is a functionally relevant measure of the size of a synapse (Morales et al., 2013). In addition, the visualization of each 3D reconstructed synapse allowed us to determine the synaptic morphology. Based on the presence of perforations or indentations in their perimeters, the synapses could be classified into four types: macular (with a flat, disk-shaped PSD), perforated (with one or more holes in the PSD), horseshoe (with an indentation in the perimeter of the PSD) or fragmented (with two or more physically discontinuous PSDs; for detailed description, see Domínguez-Álvaro et al., 2019).

To identify the postsynaptic targets of the synapses, we navigated the image stack using EspINA to determine whether the postsynaptic element was a dendritic spine (“spine” or “spines,” for simplicity) or a dendritic shaft (Fig. 5; Movies 1, 2). Unambiguous identification of spines as postsynaptic targets requires the spine to be visually traced to the parent dendrite, yielding what we refer to as fully reconstructed spines. Additionally, when synapses were established on a spine head-shaped postsynaptic element whose neck could not be followed to the parent dendrite, or whose neck was truncated, we identified these elements as non-fully reconstructed spines. These non-fully reconstructed spines were identified on the basis of their size and shape, the lack of mitochondria and the presence of a spine apparatus, or because they were filled with a characteristic fluffy material (used to describe the fine and indistinct filaments present in the spines), a term coined by Peters et al. (1991; see also del Río and DeFelipe, 1995). For simplicity, we will refer to the fully reconstructed and non-fully reconstructed spines as spines, unless otherwise specified. Similarly, for the unambiguous identification of dendritic shafts, it is necessary to be able to visually trace them inside the stack. Accordingly, when the postsynaptic element of a synapse was close to the margins, and it was truncated by the borders of the stack, the identity of the postsynaptic target could not be determined. Therefore, the targets of synapses in each of the stacks were classified into two main categories: spines and dendritic shafts, while truncated elements that could not be safely identified were discarded. When the postsynaptic target was a spine, we further recorded the position of the synapse on the head or neck. Additionally, when the postsynaptic element was identified as a dendritic shaft, it was classified as “with spines” or “without spines.”

### Spatial distribution analysis of synapses

To analyze the spatial distribution of synapses, spatial point-pattern analysis was performed as described elsewhere (Merchán-Pérez et al., 2014). For each of the 24 different samples, we calculated three functions commonly used for spatial point pattern analysis: F, G, and K functions (for detailed description, see Blazquez-Llorca et al., 2015). Additionally, we measured the distance of each synapse to its nearest synapse, to compare control and AD samples. This study was conducted using the Spatstat package and R Project program (Baddeley et al., 2015).

### Tissue shrinkage

All measurements were corrected for the tissue shrinkage that occurs during osmication and plastic-embedding of the vibratome sections containing the area of interest, as described previously (Merchán-Pérez et al., 2009). We measured the surface area and thickness of the vibratome sections with Stereo Investigator (MBF Bioscience), both before and after they were processed for EM (Oorschot et al., 1991). The surface area after processing was divided by the value before processing to obtain an area shrinkage factor (p^2^) of 0.933. The linear shrinkage factor for measurements in the plane of the section (p) was therefore 0.966. The shrinkage factor in the *z*-axis was 0.901. In addition, the total volume was corrected for the presence of fixation artifacts, which did not affect the accurate identification and quantitation of synapses (i.e., swollen neuronal or glial processes). The volume occupied by these artifacts was calculated applying the Cavalieri principle (Gundersen et al., 1988) and was discounted from the volume of the stacks of images to avoid underestimation of the number of synapses per volume. Specifically, a stereological grid with an associated area per point of 400,000 nm^2^ was superimposed onto each FIB/SEM stack using ImageJ Stereology Toolset (Mironov, 2017). Estimations were made every 20th section in each of the stacks. Every FIB/SEM stack was examined and the volume artifact ranged from 1% to 16% of the volume stacks. Volume fraction estimation was performed by point counting using the Cavalieri principle (Gundersen et al., 1988), in a similar fashion to the volume fraction estimation of cortical elements in semithin sections (see above, Volume fraction estimation of cortical elements).

All parameters measured were corrected to obtain an estimate of the preprocessing values. The shrinkage factor was used to correct the SAS area and perimeter data, while both the shrinkage and the fixation artifact factors were used to correct synaptic density values.

### Statistical analysis

To determine possible differences between the control and AD samples, statistical comparisons of the following parameters were conducted using the unpaired Mann–Whitney (MW) nonparametric *U* test: cortical thickness, synaptic density, type of synapse (AS or SS), SAS size, synaptic shape, postsynaptic targets, and distance to the nearest neighboring synapse. To determine possible differences between control and AD samples regarding volume fractions (neuronal and glial cell bodies, blood vessels and neuropil), parametric *t* test were conducted. Frequency distribution analyses were performed using Kolmogorov–Smirnov (KS) nonparametric test.

To perform statistical comparisons of AS and SS proportions, χ^2^ test was used for contingency tables. The same method was used to study whether there were significant differences between groups in relation to the shape of the synaptic junctions and their postsynaptic target. In all the χ^2^ statistical analyses, we firstly performed an “omnibus test” based on 2 × 4 contingency tables. To further investigate the specific cells driving the significance of the χ^2^ test, a partitioning procedure was applied to create 2 × 2 contingency tables (Sharpe, 2015).

Statistical analyses were performed using the GraphPad Prism statistical package (Prism 8.4.2 for Windows, GraphPad Software Inc.) and SPSS (IBM SPSS Statistics v24, IBM Corp.).

### Availability of data and materials

Most data generated or analyzed during this study are included in the main text and the tables. The control datasets used and analyzed are available on the EBRAINS Knowledge Graph (doi: 10.25493/3GMJ-FEZ). Datasets used during the current study available from the corresponding author on reasonable request.

## Results

Data regarding the synaptic organization of the human Layers II and III from the EC in the four control subjects has been previously published and detailed information can be found therein (Domínguez-Álvaro et al., 2021). What follows are the alterations of synapses in AD cases in comparison with control cases.

### Cortical thickness

Measurements of the cortical thickness were derived from semi thin toluidine blue (serial sections) using light microscopy. The mean cortical thickness of the EC was 2.0 ± 0.17 mm (mean ± SD) in the control group and 1.6 ± 0.49 mm in the cases with AD. Although ∼20% lower EC cortical thickness in AD cases was observed, we did not find a statistically significant difference between the two groups (MW, *p* = 0.34; Extended Data Table 1-1).

10.1523/ENEURO.0504-20.2021.tab1-1 Extended Data Table 1-1 EC cortical thickness in control and Alzheimer cases. All data are corrected for shrinkage factor. Download Table 1-1, DOCX file.

### Volume fraction of cortical elements

**Figure 6.**
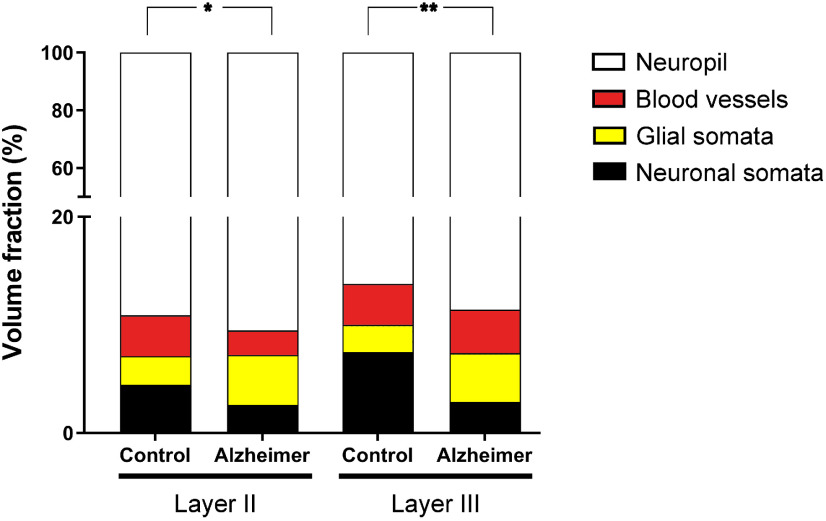
Graph showing the Vv occupied by neuronal and glial somata, blood vessels, and neuropil in both Layers II and III of the EC from control subjects and AD cases. Asterisks show the differences between groups. In Layer II, a significantly higher Vv of glial somata was found in AD cases (*t* test, *p* = 0.03). In Layer III, a significantly lower Vv of neuronal somata was found in AD cases (*t* test, *p* = 0.007). Mean values and case values are detailed in Extended Data Table 1-2, Table 1-3.

In the control group Layer II, the values of the estimated Vv occupied by neuronal somata, glial somata, blood vessels, and neuropil were 4.5%, 2.6%, 3.8%, and 89.1%, respectively. In the group with AD, these values were 2.6%, 4.6%, 2.3% and 90.5%, respectively. The only statistically significant difference was that of the Vv of glial somata, which was found to be significantly higher in cases with AD (*t* test, *p* = 0.03; Fig. 6; Extended Data Table 1-2, Table 1-3).

10.1523/ENEURO.0504-20.2021.tab1-2 Extended Data Table 1-2 Volume fraction occupied by cortical elements in Layers II and III of the EC. Data are given as mean ± SD; Vneu, volume fraction occupied by neurons; Vg, volume fraction occupied by glia; Vbv, volume fraction occupied by blood vessels; Vn, volume fraction occupied by neuropil. Data from individual cases are shown in Extended Data Table 1-3. Download Table 1-2, DOCX file.

10.1523/ENEURO.0504-20.2021.tab1-3 Extended Data Table 1-3 Light microscopy data on volume fraction occupied by cortical elements in Layers II and III of the EC for individual cases. All volume data are corrected for shrinkage. Vneu, volume fraction occupied by neurons; Vg, volume fraction occupied by glia; Vbv, volume fraction occupied by blood vessels; Vn, volume fraction occupied by neuropil. Download Table 1-3, DOCX file.

In Layer III of the control group, the Vv of neuronal somata, glial somata, blood vessels, and neuropil were 7.5%, 2.5%, 3.8%, and 86.2%. In cases with AD, these values were 2.9%, 4.6%, 4.0%, and 88.6%. Statistical comparisons showed a significantly lower Vv of neuronal somata (*t* test, *p* = 0.007) in AD (Fig. 6; Extended Data Table 1-2, Table 1-3).

### Synaptic density and AS:SS ratio

A total of 2019 synapses from AD cases (1044 synapses in Layer II and 1065 in Layer III) were identified and reconstructed in 3D, after discarding incomplete synapses or those touching the exclusion edges of the CF, and the total volume analyzed was 9006 μm^3^ in AD samples (Table 2; Extended Data Table 2-1).

**Figure 7.**
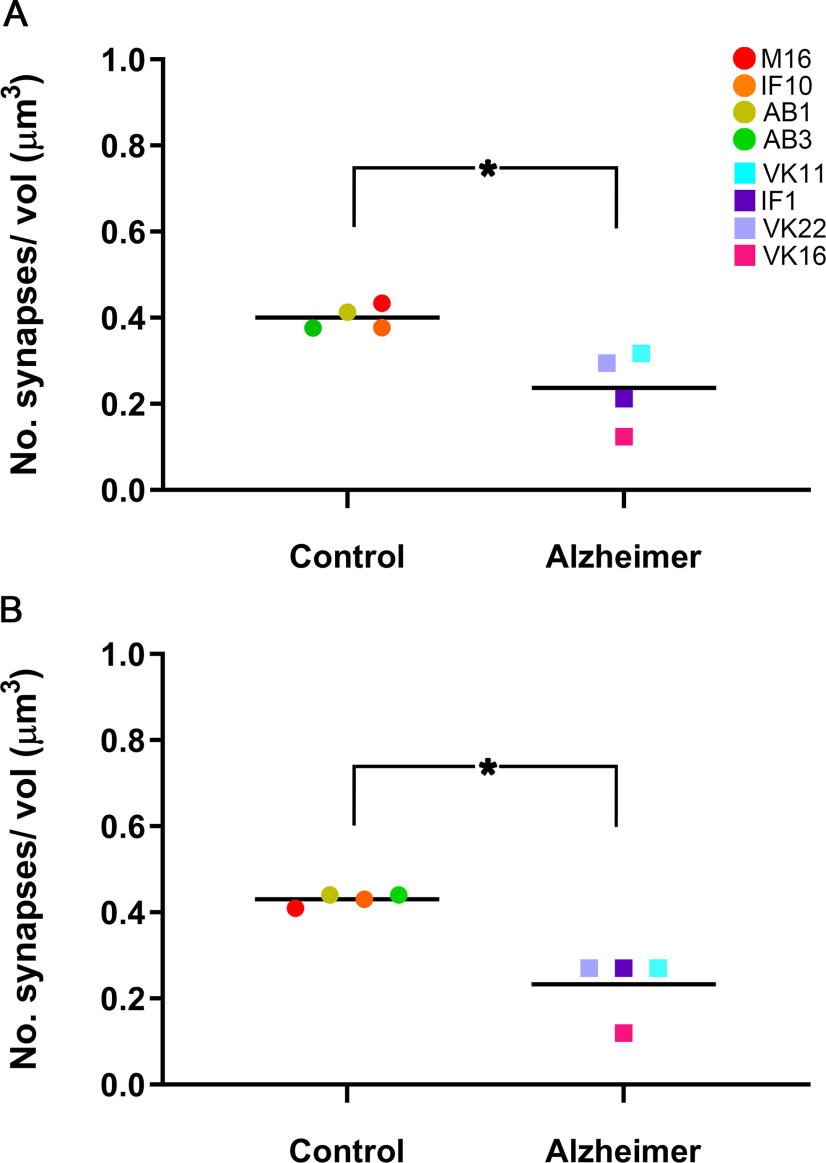
Graphs showing the overall mean synaptic density in Layer II (***A***) and Layer III (***B***) of the EC in control and AD cases. Control cases are represented by circles and AD cases are represented by squares. Each color corresponds to each case analyzed, as denoted in the upper right-hand corner. Asterisks show significant differences between groups: in both Layers II and III of the EC, the mean synaptic density was significantly lower in AD cases (MW, *p* = 0.03).

A significantly lower synaptic density (taking both AS and SS together) was observed in AD cases, this value was 40% lower than in control cases in both EC layers. In Layer II, we found 0.24 synapses/μm^3^ in AD cases and 0.40 synapses/μm^3^ in control cases (MW, *p* = 0.03; Fig. 7*A*). In Layer III, the mean synaptic density in AD was 0.24 synapses/μm^3^ compared with 0.43 synapses/μm^3^ found in control cases (MW, *p* = 0.03; Fig. 7*B*; Table 2; Extended Data Table 2-1).

In Layers II and III, the AS:SS ratio for the AD cases was very similar to that of the control cases (close to 92:8 in both layers and cases; Table 2; Extended Data Table 2-1), and no statistically significant differences were found between the control group and the AD group in any layer (χ^2^, *p* > 0.05).

### 3D spatial synaptic distribution

Analysis indicates a clear fit to a complete spatial randomness (CSR) model, since F, G, and K functions closely resemble the theoretical curve that these functions represent, both in the control and the AD cases. The CSR model defines a situation where a point is equally likely to occur at any location within the study volume, regardless of the locations of other points. Therefore, a CSR model is considered as a reference for a random pattern in spatial point process statistics (for review, see Merchán-Pérez et al., 2014). That is, the spatial distribution of the synapses fitted a random distribution in all subjects and layers. Furthermore, the estimation of the distance from each synapse to its closest synapse showed that although the mean distance was greater in AD cases in both Layer II (840 nm in control; 938 nm in AD; Table 2; Extended Data Table 2-1) and Layer III (852 nm in control, 1004 nm in AD; Table 2; Extended Data Table 2-1), no statistically significant differences were found (MW, *p* > 0.05).

### Study of the synapse characteristics

#### Synaptic size

The AD samples did not show differences between layers in the SAS area of AS (MW, *p* = 0.41; Table 3; Extended Data Table 3-1). However, we found significantly larger mean values of SAS area for AS (MW, *p* = 0.03) from Layer II in AD cases compared with the controls. These differences were not found in SS or in Layer III (MW, *p* > 0.05; Table 3; Extended Data Table 3-1).

**Table 4:** Proportion of the different synaptic shapes in Layers II and III of the EC

EC layer	Group	Type of synapse	Macular synapses	Perforated synapses	Horseshoe synapses	Fragmented synapses	Total synapses
II	Control	AS	83.0% (1285)	12.9% (200)	3.3% (51)	0.8% (13)	100% (1549)
		SS	73.0% (100)	11.0% (15)	15.3% (21)	0.7% (1)	100% (137)
	Alzheimer	AS	80.6% (769)	13.4% (128)	4.9% (47)	1.1% (10)	100% (954)
		SS	73.4% (66)	12.2% (11)	12.2% (11)	2.2% (2)	100% (90)
III	Control	AS	75.9% (1326)	19.9% (347)	3.3% (58)	0.9% (15)	100% (1746)
		SS	65.9% (85)	15.5% (20)	17.0% (22)	1.6% (2)	100% (129)
	Alzheimer	AS	82.8% (806)	10.2% (99)	4.3% (42)	2.7% (26)	100% (973)
		SS	73.9% (68)	8.7% (8)	10.9% (10)	6.5% (6)	100% (92)

Data are given as percentages with the absolute number of synapses studied in parentheses. Data for individual cases are shown in Extended Data Table 4-1, Table 4-2.

10.1523/ENEURO.0504-20.2021.tab4-1 Extended Data Table 4-1 Proportion of macular, perforated, horseshoe-shaped, and fragmented synapses in Layer II of the EC for individual cases. Data are given as percentages with the absolute number of synapses studied in parentheses. Download Table 4-1, DOCX file.

10.1523/ENEURO.0504-20.2021.tab4-2 Extended Data Table 4-2 Proportion of macular, perforated, horseshoe-shaped, and fragmented synapses in Layer III of the EC for individual cases. Data are given as percentages with the absolute number of synapses studied in parentheses. Download Table 4-2, DOCX file.

**Figure 8.**
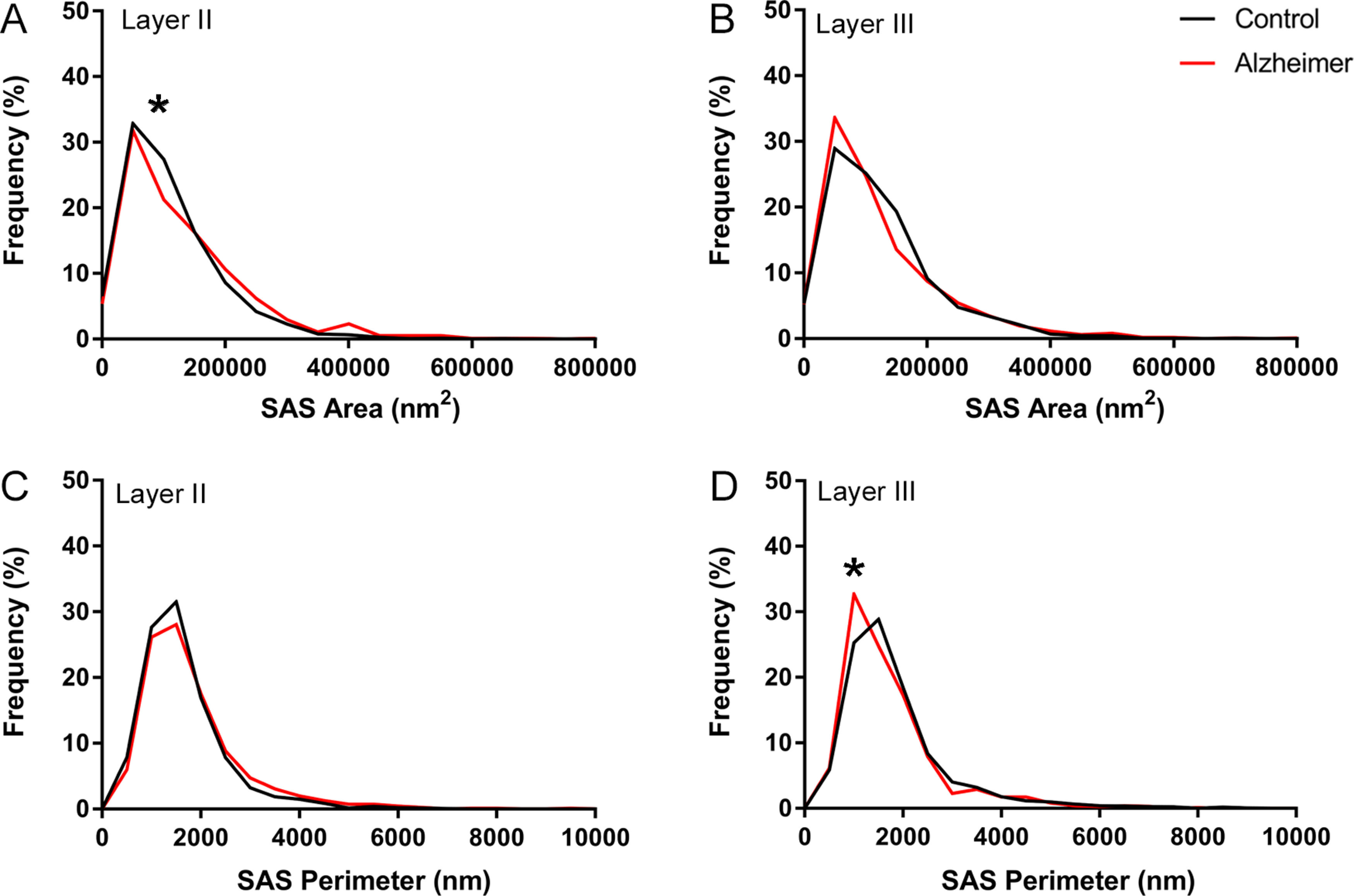
Graph showing the frequency distribution plots of AS SAS area (***A***, ***B***) and perimeter (***C***, ***D***), in Layers II and III of the EC in control subjects (black) and AD cases (red). Statistical comparisons between groups showed significant differences (KS, *p* < 0.0001; indicated with an asterisk) in the frequency distributions of SAS area in Layer II (***A***), and in the frequency distribution of SAS perimeter in Layer III (***D***).

Analysis of the frequency distributions of the area and perimeter of the AS showed significant differences in the frequency distributions of area in Layer II and in the perimeter in Layer III (KS, *p* < 0.0001), indicating a lower proportion of small SAS area in Layer II in AD cases and higher proportions of small SAS perimeter in Layer III in AD cases (Fig. 8).

#### Synaptic shape

**Figure 9.**
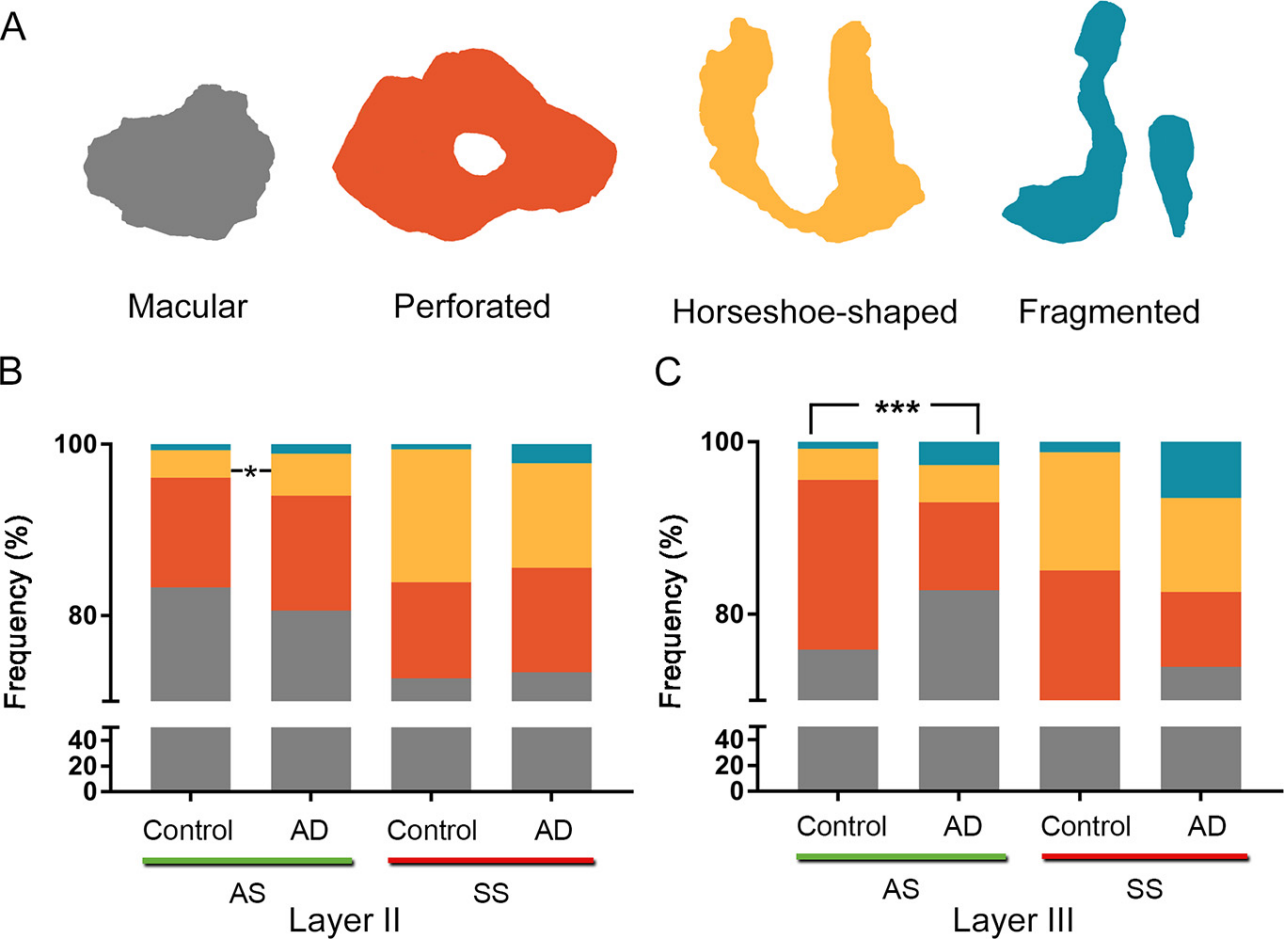
Proportions of the different synaptic shapes (***A***) in Layers II (***B***) and III (***C***) of the EC in control subjects and AD cases. ***A***, Schematic representation of the synaptic shapes: macular synapses, with a continuous disk-shaped PSD; perforated synapses, with holes in the PSD; horseshoe-shaped, with a tortuous horseshoe-shaped perimeter with an indentation; and fragmented synapses, with two PSDs with no connections between them. Proportions of macular, perforated, horseshoe-shaped, and fragmented AS and SS are displayed for Layers II (***B***) and III (***C***) of the EC in control subjects and AD cases. Statistical differences (indicated with asterisks) showed that in Layer II, horseshoe-shaped AS were more frequent (χ^2^, *p* = 0.04) in AD cases; and in Layer III of the AD group, fragmented (χ^2^, *p* = 0.0004) and macular (χ^2^, *p* < 0.0001) AS were more frequent, whereas perforated AS were less frequent in AD cases than in control individuals (χ^2^, *p* <0.0001).

Most synapses, AS and SS, presented a macular shape in both control and AD cases in Layers II and III (>80%), while synapses with more complex shapes (i.e., horseshoe-shaped, perforated, or fragmented) were less frequent (Fig. 9; Table 4; Extended Data Table 4-1, Table 4-2). Evaluation of the possible differences between the control and the AD group in Layer II showed a slightly higher proportion of horseshoe-shaped AS in AD samples (χ^2^, *p* = 0.04; Table 4; Extended Data Table 4-1; Fig. 9*B*). Analysis of Layer III revealed that fragmented (χ^2^, *p* = 0.0004) and macular (χ^2^, *p* < 0.0001) AS were more frequent in the AD group, whereas perforated AS were less frequent in AD cases than in the control individuals (χ^2^, *p* <0.0001; Table 4; Extended Data Table 4-2; Fig. 9*C*). Analysis of the SS in Layers II and III did not reveal differences between groups (χ^2^, *p* > 0.001).

**Table 5:** Distribution of AS and SS on spines and dendritic shafts in Layers II and III of the EC

Layer	Group	Type of synapse	On spine heads	On nf. spines	On spine necks	On aspiny dendritic shaft	On spiny dendritic shaft	Total
II	Control	AS	39.68% (567)	18.89% (270)	0.56% (8)	19.87% (284)	20.99% (300)	100% (1429)
		SS	9.38% (12)	4.69% (6)	0.78% (1)	22.66% (29)	62.50% (80)	100% (128)
	Alzheimer	AS	35.19% (284)	17.84% (144)	0.87% (7)	25.15% (203)	20.94% (169)	100% (807)
		SS	10.98% (9)	1.22% (1)	0% (0)	48.78% (40)	39.02% (32)	100% (82)
III	Control	AS	39.68% (642)	15.33% (248)	0.74% (12)	17.49% (283)	26.79% (433)	100% (1618)
		SS	11.90% (15)	4.76% (6)	2.38% (3)	22.22% (28)	58.73% (74)	100% (126)
	Alzheimer	AS	41.67% (305)	11.75% (86)	0.82% (6)	28.83% (211)	16.94% (124)	100% (732)
		SS	5.88% (5)	1.18% (1)	3.53% (3)	40% (34)	49.41% (42)	100% (85)

Synapses on spines have been subdivided into those that are established on spine heads and those that are established on spine necks. Moreover, we differentiated between aspiny and spiny dendritic shafts. Data are expressed as percentages with the absolute number of synapses studied given in parentheses. Data for each individual case are shown in Extended Data Table 5-1, Table 5-2. nf.: non-fully reconstructed.

10.1523/ENEURO.0504-20.2021.tab5-1 Extended Data Table 5-1 Distribution of AS and SS on spines and dendritic shafts in Layer II of the EC for individual cases. Synapses on spines have been subdivided into those that are established on spine heads and those that are established on spine necks. Synapses on spine have been recorded separately. Moreover, we differentiated between aspiny and spiny dendritic shafts. Data are expressed as percentages with the absolute number of synapses studied given in parentheses. Download Table 5-1, DOCX file.

10.1523/ENEURO.0504-20.2021.tab5-2 Extended Data Table 5-2 Distribution of AS and SS on spines and dendritic shafts in Layer III of the EC for individual cases. Synapses on spines have been subdivided into those that are established on spine heads and those that are established on spine necks. Synapses on spines have been recorded separately. Moreover, we differentiated between aspiny and spiny dendritic shafts. Data are expressed as percentages with the absolute number of synapses studied given in parentheses. Download Table 5-2, DOCX file.

#### Analysis of synaptic size and shape

Additionally, we determined whether the shape of the synapses was related to their size. For this purpose, the area and perimeter of the SAS of AS were analyzed according to their synaptic shape (SS were not analyzed since the sample size was too small).

When we compared the synaptic size considering the synaptic shape, we found that the mean SAS area from perforated AS in Layer III was larger in AD samples (296,156 ± 24,116 nm^2^, mean ± SEM) than in the controls (228,057 ± 6993 nm^2^, mean ± SEM; MW, *p* = 0.03; Extended Data Tables 4-3, 4-4). No differences were found in the mean area and perimeter of the SAS from macular, horseshoe-shaped, and fragmented synapses (MW, *p* > 0.05). Analysis of the frequency distribution of the area and perimeter showed no differences between groups (Extended Data Fig. 9-1; KS; *p* > 0.0001).

10.1523/ENEURO.0504-20.2021.tab4-3 Extended Data Table 4-3 Area (nm^2^) and perimeter (nm) of the macular, perforated, horseshoe-shaped, and fragmented SAS in Layer II of the EC. All data are corrected for shrinkage. Download Table 4-3, DOCX file.

10.1523/ENEURO.0504-20.2021.tab4-4 Extended Data Table 4-4 Area (nm^2^) and perimeter (nm) of the macular, perforated, horseshoe-shaped, and fragmented SAS in Layer III of the EC. All data are corrected for shrinkage factor. Download Table 4-4, DOCX file.

10.1523/ENEURO.0504-20.2021.f9-1 Extended Data Figure 9-1 Frequency histograms of the SAS area (***A***, ***B***, ***E***, ***F***) and perimeter (***C***, ***D***, ***G***, ***H***) of macular, perforated, horseshoe-shaped, and fragmented AS from control cases (***A***, ***C***, ***E***, ***G***) and from AD cases (***B***, ***D***, ***F***, ***H***), in Layers II and III of the EC. The SAS area and perimeter of macular synapses were significantly smaller than in perforated, horseshoe-shaped, and fragmented synapses (KW, *p* < 0.0001). No differences were found between control and AD cases (KS, *p* > 0.0001). Download Figure 9-1, TIF file.

### Study of the postsynaptic targets

The postsynaptic targets of 1706 synapses were determined from the AD samples. In Layer II, we identified the postsynaptic targets of a total of 807 AS and 82 SS (Table 5; Extended Data Table 5-1), and in Layer III, the postsynaptic targets for 732 AS and 85 SS were determined (Table 5; Extended Data Table 5-2).

**Figure 10.**
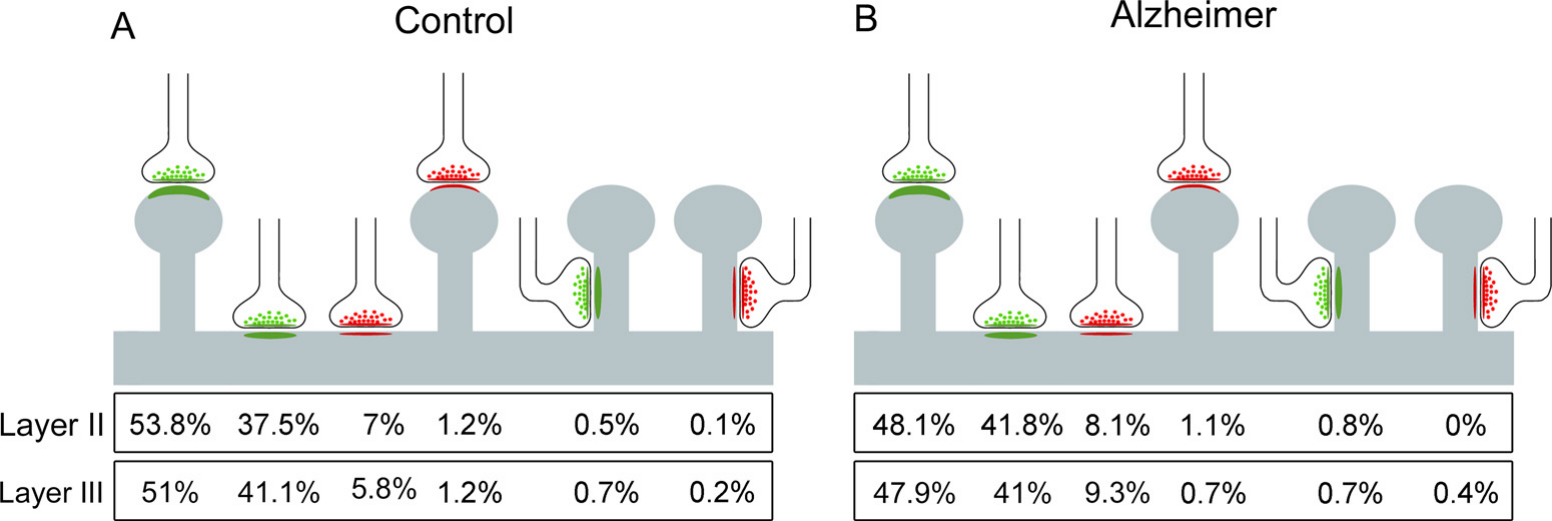
Postsynaptic target distribution in Layers II and III of the EC. Schematic representation of the distribution of AS and SS on different postsynaptic targets from control cases (***A***) and AD cases (***B***). In both Layers II and III, the AS preferentially targeted spine heads, while the SS preferentially targeted dendritic shafts. Percentages of postsynaptic targets are indicated, showing, from left to right, the most frequent type (AS on spine heads) to the least frequent type (SS on spine necks). Synapses on spines have been subclassified into those that are established on the spine head and those established on the neck.

The most abundant type of synapse in AD individuals was AS on spine heads (range 47.9–48.1%) closely followed by AS on dendritic shafts (41−41.8%). SS on dendritic shafts were less common (8.1−9.3%), SS on spine heads were very uncommon (0.7–1.1%), and AS or SS on spine necks were very infrequent indeed (<1%; Fig. 10).

When the preference of the synaptic types (AS or SS) for a particular postsynaptic element was analyzed, we found that, in both controls and AD individuals, excitatory contacts (AS) presented a statistically significant preference for spines, whereas inhibitory contacts (SS) preferentially targeted dendritic shafts (χ^2^, *p* < 0.001), in both layers, in line with the findings in control EC samples from the same layers (Domínguez-Álvaro et al., 2021). Contingency tables were used to assess possible differences in the distribution of postsynaptic elements of cases with AD. No statistically significant differences were found (χ^2^, *p* > 0.001), although a slightly lower proportion of AS established on spine heads (χ^2^, *p* = 0.02) and a higher proportion of AS on dendritic shafts were observed in AD samples from Layer II (χ^2^, *p* = 0.03).

We also analyzed whether there was a relationship between the synaptic size and the type of postsynaptic element. When we compared the synaptic size of AS associated with the postsynaptic targets between control cases and AD cases, no differences were found in any layer with regard to the area and perimeter of the SAS (MW, *p* > 0.05) or their frequency distribution (KS, *p* > 0.001; Extended Data Fig. 10-1).

10.1523/ENEURO.0504-20.2021.f10-1 Extended Data Figure 10-1 Frequency histograms of the SAS area (***A***, ***B***, ***E***, ***F***) and perimeter (***C***, ***D***, ***G***, ***H***) of AS targeting spine heads, spine necks and dendritic shafts from control cases (***A***, ***C***, ***E***, ***G***) and from AD cases (***B***, ***D***, ***F***, ***H***), in both Layers II and III of the EC. No differences were found between groups (KS, *p* > 0.0001). Download Figure 10-1, TIF file.

## Discussion

In total, there are four main findings in the present study on the human EC: at the light microscope level, (1) a significantly lower volume fraction occupied by neuronal bodies in the Layer III was found in AD cases; at the ultrastructural level, (2) a significantly lower synaptic density was found in both layers in AD cases; (3) synaptic morphology analysis revealed larger and more complex synapses in Layer II in AD cases; and (4) there was a greater proportion of small and simple synapses in Layer III in AD cases than in control individuals.

### Cortical thickness and volume fraction analysis

At the light microscopic level, atrophy of the EC and adjacent cortical regions is clearly visible to the naked eye (Fig. 1). However, the difference in the mean cortical thickness was no evident (Extended Data Table 1-1). Macroscopic atrophy of the EC has been described in AD cases (Van Hoesen et al., 1991). This cortical atrophy and, more specifically, the neuronal loss (see below) observed in AD cases have been related to the presence of NFTs and Aβ plaques, with reports indicating an inverse relationship between the number of neurons and the degree of neuropathology (Van Hoesen et al., 1991; Gómez-Isla et al., 1996).

In AD brain samples, we found a significant lower neuronal volume fraction in the Layer III. This is in line with the reported neuronal loss from AD cases in the EC (Van Hoesen and Hyman, 1990; Gómez-Isla et al., 1996; Šimić et al., 2017). Since the neurons of EC Layer III constitute the projection elements of the perforant pathway to the hippocampal CA1 (together with neurons from Layer II), this neuronal loss may contribute to the memory impairment described in AD cases (Hyman et al., 1986; Van Hoesen et al., 1991; von Gunten et al., 2006; Andrade-Moraes et al., 2013). Since our present neuronal volume fraction estimations are based on semithin sections, and different types of neurons have not been identified, we cannot rule out a selective neuronal loss that may affect either to excitatory neurons or to GABAergic interneurons or to both cell types.

The significantly higher volume fraction occupied by glial somata found in Layer II is in line with previous reports showing a larger density of glial cells in human EC from AD cases (Muramori et al., 1998). However, no significant differences were found in Layer III, suggesting a layer-selective change. The effects of gliosis during AD development are still under debate. It has been proposed that gliosis mediates an inflammatory response to prevent the progression of the disease but that this response may in fact contribute to neurodegeneration (for review, see Fakhoury, 2018).

### Synaptic changes in AD

#### Synaptic density, proportions, and spatial distribution

Our main finding was a significantly lower synaptic density (around 40% lower) in the neuropil of both Layers II and III of AD individuals compared with controls.

In brain tissue samples from individuals with AD, a lower density of synapses has been described compared with controls in numerous cortical regions, including the DG and certain frontal, parietal, temporal and cingulate cortical areas (Scheff et al., 1990, 1996; Scheff and Price, 1993, 1998, 2003, 2006). A decrease in the total number of synapses, accompanied by brain atrophy, has been also reported in CA1, the DG and some cingulate and temporal cortices (Scheff et al., 2006, 2007, 2011, 2015; Montero-Crespo et al., 2021). However, other studies have not found differences in synaptic density in layers III and V of EC samples from AD cases (Scheff et al., 1993). These discrepancies may be because of differences in the methodologies used. Furthermore, numerous studies have estimated the synaptic density using indirect methods, either using light microscopy to count immunoreactive puncta for synaptic markers (Masliah et al., 1990; Honer et al., 1992), or employing conventional transmission EM to examine single or few serial ultrathin sections (Scheff et al., 1993). Quantification of synaptic density in single ultrathin sections using transmission EM to infer 3D characteristics of synaptic junctions observed in two dimensions may lead to inaccurate synaptic density estimations (see Merchán-Pérez et al., 2009). Thus, the present results revealing a lower number of synapses per volume of neuropil, along with the atrophy of the EC compared with controls, suggest a dramatic decrease in the absolute number of synapses in AD cases in these EC layers.

Synaptic loss has been widely reported as the characteristic that best correlates with cognitive deficit in AD cases (for review, see Colom-Cadena et al., 2020). Synaptic loss occurs in the early stages of the disease, affecting firstly the subcortical regions and the EC, and progressing to other cortical regions (Braak and Braak, 1991; Braak and Del Tredici, 2012). Tau protein and β-amyloid peptide in pathologic conditions could have toxic effects on synapses leading to synaptic loss and/or dysfunction in AD (Zhou et al., 2017; for review, see Henstridge et al., 2016; Rajmohan and Reddy, 2017). This synaptic loss and impairment may cause dysfunction in the cortical circuits contributing to the decline in cognition (for review, see Colom-Cadena et al., 2020).

Previous studies have shown that the percentage of AS and SS varies, 80–95% and 20–5%, respectively, in all the cortical layers, cortical areas and species examined so far by transmission EM (Beaulieu and Colonnier, 1985; Megías et al., 2001; Bourne and Harris, 2011; DeFelipe, 2011, 2015). Estimations with samples from human brain using the same method have shown that the AS:SS ratio in Layer III of the temporal area 21 is 93:7 (Cano-Astorga et al., 2021), 96:4 in the transentorhinal cortex (Domínguez-Álvaro et al., 2018), and 95:5 in the human CA1 hippocampal field (except in the stratum lacunosum moleculare, in which this ratio was 89:11; Montero-Crespo et al., 2020). Thus, it would appear that the present AS:SS ratio data provide among the highest and lowest proportions previously observed in different brain regions, for AS and SS, respectively. From a functional point of view, the proportion of excitatory and inhibitory synapses is critical, since higher or lower proportions are linked to differences in the excitatory/inhibitory balance of the cortical circuits (for review, see Froemke, 2015; Zhou and Yu, 2018; Sohal and Rubenstein, 2019). Interestingly, no changes in the proportions of AS and SS were observed; therefore, the lower synaptic density in AD cases might affect AS and SS equally, which is in line with our previous results in the transentorhinal cortex (Domínguez-Álvaro et al., 2018). Since most of the synapses are excitatory synapses (∼92% AS), the synaptic decrease may result in a massive loss of AS in particular. As the present results did not reveal differences in the AS:SS ratio between control and AD cases in the neuropil in any layer, it could be interpreted that the proportion of AS and SS on the dendritic arbor of the different types of neurons may be similar. However, it has been shown that there are differences in the number of GABAergic and glutamatergic synaptic inputs in different neuronal types in other cortical regions of a variety of species (DeFelipe and Fariñas, 1992; Freund and Buzsáki, 1996; DeFelipe, 1997; Somogyi et al., 1998; Schubert et al., 2007; Markram et al., 2015; Tremblay et al., 2016; Hsu et al., 2017). Thus, it would be necessary to examine the synaptic inputs on each specific neuronal type to determine actual differences in the AS:SS ratio in particular cell types, although the final general AS:SS ratio does not vary in the neuropil.

Furthermore, it has been reported that GABA inhibitory interneurons in the brains of AD cases are abnormally reduced, along with the inhibitory neurotransmitters (for review, see Xu et al., 2020). Other studies have shown that somatostatin neurons, calretinin neurons, and parvalbumin neurons are decreased in several brain regions including the EC of AD cases (Solodkin and Van Hoesen, 1996; Mikkonen et al., 1999). Therefore, further studies should be performed to examine the GABAergic innervation of neurons in the EC.

Finally, the present results indicate that the distribution of synapses in the neuropil of both control and AD samples is nearly random, only constrained by the fact that synapses cannot physically overlap in space and so their geometric centers or centroids cannot be too close to their neighbors. As we have previously shown in human brain samples from hippocampal CA1, EC, transentorhinal cortex and temporal cortex, there seems to be no limitation to the position of any synapse except the space already occupied by other synapses (Domínguez-Álvaro et al., 2018, 2021; Montero-Crespo et al., 2020; Cano-Astorga et al., 2021). Previous studies in the plaque-free neuropil of AD cases also showed a random distribution pattern of the synapses in other brain regions (Blazquez-Llorca et al., 2013; Domínguez-Álvaro et al., 2018; Montero-Crespo et al., 2021). It has been reported that during cortical development, synapses are randomly added and/or withdrawn from the population, as described by the synaptic turnover (Rakic et al., 1994). It seems that the only constraint for a synapse to form is that this particular spot is not already occupied by a preexisting synapse (for review, see Merchán-Pérez et al., 2014). Interestingly, in the AD cases, the loss of synapses per unit volume does not affect their 3D distribution.

#### Shape and size of the synapses

There are very few studies on human brain at the ultrastructural level that provide 3D data on the morphologic characteristics of the synaptic junctions to compare with our findings. In the examined EC layers, excitatory AS were larger than inhibitory SS, as previously shown in human transentorhinal and temporal cortex (Domínguez-Álvaro et al., 2018; Cano-Astorga et al., 2021) and in several strata from the hippocampal CA1 (Montero-Crespo et al., 2020). Furthermore, no differences between layers were found regarding the SAS area of AS in AD cases (136,166 nm^2^ in Layer II and 122,727 nm^2^ in Layer III), which was in contrast with previous results in control EC showing larger synapses in Layer III (Domínguez-Álvaro et al., 2021). However, the synaptic size in AD samples differs from that of control individuals, with larger and more complex synapses in Layer II and a greater proportion of smaller synapses in Layer III.

Several EM studies conducted with samples of human brain tissue have reported an increase in the size of synaptic apposition, and this has been suggested as a possible compensatory mechanism for the synaptic loss (for review, see Scheff and Price, 2006). Models studying the relationship between the size of synaptic junctions and the probability of neurotransmitter release suggest that larger synapses (with a greater number of postsynaptic receptors, mainly AMPA receptors) may produce more powerful, homogeneous and numerous synaptic responses, while smaller synapses (with fewer receptors, mainly NMDA) may produce weaker and more variable responses (Kharazia and Weinberg, 1999; Montes et al., 2015). Thus, alterations in the normal size of the synaptic junction may change the proportion of postsynaptic receptors, thereby modifying the synaptic response.

Regarding synaptic shape, we found a higher proportion of horseshoe synapses in Layer II from AD cases. In Layer III from these AD individuals, higher proportion of AS with macular and fragmented shape were found along with a lower proportion of perforated AS synapses. Several studies have linked an increase in synaptic complexity to an increase in the efficiency of synaptic transmission, via the insertion of new receptors in the postsynaptic membrane (Lüscher et al., 2000). Perforated synapses have higher immunoreactivity for glutamate receptors than non-perforated synapses (Ganeshina et al., 2004a,b). The larger proportion of macular AS in Layer III (around 83% in AD cases, vs 76% in control cases) and the corresponding lower number of perforated synapses (about half the number found in control cases) supports the evidence for a larger number of simple synapses in this layer in AD cases, which is in turn supported by the finding of a higher proportion of smaller synapses described above. However, the higher proportion of fragmented AS in Layer III from AD cases may indicate the above-mentioned compensatory response to the synaptic loss. Conversely, small macular synapses from Layer III may be more susceptible to damage resulting in a reduction in their numbers. That is, synaptic morphology may be selectively altered by layer, with a non-homogenous pattern of changes, which would differentially affect the circuits in which these layers are involved.

Therefore, synaptic changes in Layers II and III might be part of a remodeling process of the remaining synapses, which overcomes the overall synaptic loss. If the remodeling process involves an increase in the proportion of synapses with more complex morphology, it could be hypothesized that the morphologic changes in AD would trigger compensatory mechanisms during the progression of the disease. However, this type of compensatory mechanism may fail or be deficient, leading to a decline in the synaptic functionality accompanying the progression of the disease.

### Postsynaptic targets

Despite the lower synaptic density in AD cases, no changes in the distribution and types of postsynaptic targets were found. In the AD samples, the most frequent combinations were axospinous AS (on dendritic spine heads) and axodendritic AS (established on dendritic shafts). In addition, the majority of the synapses that a pyramidal cell receives are on spines and they represent the vast majority of AS synapses (DeFelipe and Fariñas, 1992). Using the same FIB/SEM technology, in Layer II of the human transentorhinal cortex, it was reported that 60% of all AS were established on spines that were fully reconstructed (Domínguez-Álvaro et al., 2019). However, when we re-analyzed the stacks of images of the TEC to include the non-fully reconstructed spines, the AS percentage established on dendritic spine heads was 75%, which is similar to the case of the AS found in Layer III of the temporal cortex (75%) using the same analysis method (Cano-Astorga et al., 2021). This percentage was much higher in the CA1 hippocampal field, as high as 94% in the superficial part of the CA1 stratum pyramidale (Montero-Crespo et al., 2020). In both Layers II and III of the EC, the percentage of AS on spines was 57%, and 43% in the case of AS on dendritic shafts. This is a higher proportion of AS on shafts than in the other human cortical regions previously analyzed using the same techniques.

In addition, the comparison between control and AD samples did not show a clear reduction in the proportion of AS on spines. Only a slightly lower proportion of AS established on spines was found in Layer II from AD samples. Therefore, the lower synaptic density does not seem to be specifically related to a loss of spines, but rather a general loss of synapses might occur regardless of their postsynaptic targets. It may be that the axospinous synapses in EC from AD cases are functionally altered although no changes were found in their proportions.

### Implications of the synaptic changes

EC provides excitatory inputs to the hippocampus from Layer II neurons targeting the DG (trisynaptic pathway) and from Layer III neurons monosynaptically targeting CA1 (for review, see Insausti and Amaral, 2012; Marks et al., 2020). Neuronal projections from both layers give rise to the perforant pathway, which has generally been reported to be weakened in AD (Hyman et al., 1986).

Moreover, in Layer II there are two subpopulations of excitatory neurons: spiny stellate neurons (which project to DG, CA3 and CA2) and modified pyramidal neurons (which project to stratum lacunosum in CA1). Projections from these neurons, constituting the trisynaptic pathway, seem to be related to contextual memories (for review, see Marks et al., 2020). It has been proposed that this pathway is more susceptible to premature degeneration than the monosynaptic pathway (Van Hoesen et al., 2006; Llorens-Martín et al., 2014).

In addition, pyramidal neurons from Layer III directly project to pyramidal neuron dendrites located in the stratum moleculare of CA1, and it has been proposed that these monosynaptic excitatory inputs drive temporal association learning (for review, see Marks et al., 2020).

The present study shows a significant lower number of synapses per volume, as well as morphologic synaptic alterations in the EC from AD cases in Layers II and III. Since the AD cases examined in the present study correspond to advanced stages of the disease, we do not know when the synaptic alterations occurred. Considering that there is neuronal loss, the lower number of synapses may be explained, at least in part, by the loss of local axons of these neurons, at least at the earlier stages of the disease. However, the lower number of synapses may also be explained by a loss of projection neurons that die in distant cortical regions that are also affected by the disease.

### Interindividual variability

The present data cannot be generalized to the whole population of patients with AD, which shows clear variability between individuals (Fig. 7; Table 2). Therefore, our study can be considered as a further step to tackle the issue of the synaptic alterations in AD cases, but it would be necessary to validate our results, both in a larger number of individuals and in additional brain regions (which we have already done in the hippocampal CA1 field and in the transentorhinal cortex). Technical effects could be ruled out given that the postmortem delays were all similar and the procedures used were the same. Moreover, all of the AD cases examined were women aged between 80 and 86 years old; therefore, variability because of sex and aging could also be ruled out.

Considering the above-mentioned toxic effect of tau protein and β-amyloid peptide on the synapses, a relationship between the degree of disease progression and the synaptic loss might be expected: the greater the degree of the pathology, the greater the synaptic loss. Subject VK16, who presented a high score for neuropathology and disease progression (Braak/CERAD: VI/C), also had the lowest synaptic density in both layers (0.12 synapse/μm^3^; Fig. 7; Extended Data Table 2-1). However, subject VK22, who also had a high score for neuropathology and disease progression (V/C), had a synaptic density of more than double (0.29 and 0.27 synapse/μm^3^, for Layers II and III, respectively) and both cases, VK16 and VK22, suffered dementia. However, subject IF1 did not present evidence of dementia despite the fact that this subject had a high level of pathology (IV/B) and low synaptic density, which was similar to VK22 (0.21 and 0.27 synapse/μm^3^, for Layers II and III, respectively). However, the cortical thickness of case IF1 was 2.3 mm, whereas in VK16 and VK22, the cortical layers were thinner (1.19 and 1.51 mm, respectively; Extended Data Table 1-1). As pointed out by Ferrer (2012), it should be kept in mind that AD, at least and limited to the entorhinal and transentorhinal cortices (Stages I–II), affects ∼80% of individuals over 65 years, but dementia only occurs in a small percentage of individuals in this age bracket (the prevalence of dementia in AD increases to 25% in 80-year-old individuals). Thus, it is possible that this particular case (IF1) may represent a predementia stage of AD (prodromal AD).

Control cases were on average younger (51 years old) than AD cases (85 years old), and it remains uncertain whether some of the synaptic alterations observed in the present work were because of normal aging or because of the pathology per se. Although no sex-based differences have been reported with aging in the human cerebral cortex (Scheff et al., 2001), previous studies on synaptic density in the human temporal cortex have shown differences depending on the sex (Alonso-Nanclares et al., 2008). Nevertheless, no similar EM studies have been performed in the EC in individuals with different ages and sexes. Thus, the lower synaptic density found in AD cases might also be the results of sex or age effects. In aged rhesus monkey, a lower number of synapses has been reported in prefrontal cortex related to a cognitive decline; however, studies in rats and monkeys have shown no evidence of synaptic loss with age in mesial temporal lobe structures (for review, see Morrison and Baxter, 2012). Further studies using human brain tissue from older control cases would be necessary to determine whether or not these differences in the synaptic density are because of these factors, as opposed to the AD per se. Nevertheless, it is important to point out that only the neuropil without plaques was examined in the present study, whereas it is well established that the neuropil which is adjacent to, and is surrounding, the plaques shows synaptic alterations (Blazquez-Llorca et al., 2013). Thus, it is possible that the neuropil lacking plaques in AD cases displays “normal” characteristics, and differences observed in our study comparing to the control group might be because of factors related to sex and age.

In the present study, we found, in addition to synaptic loss, changes in the morphology of the synapses in AD compared with control cases. These structural changes may contribute to the anatomic basis for the impairment of cognitive functions in AD. Despite the large number of synapses analyzed (5000 synapses), and the proven robustness of the methodology used in this study, it should be kept in mind that (1) the data comes from the analysis of four control cases and four AD cases and (2) we cannot rule out sex-related and age-related factors that may influence the differences observed between the two groups. In order to address these remaining issues, it would be necessary to perform further studies using FIB/SEM or other similar techniques to reconstruct thousands of synapses in aged control cases of both sexes.

## Synthesis

Reviewing Editor: Alexey Semyanov, Shemyakin-Ovchinnikov Institute of Bioorganic Chemistry Decisions are customarily a result of the Reviewing Editor and the peer reviewers coming together and discussing their recommendations until a consensus is reached. When revisions are invited, a fact-based synthesis statement explaining their decision and outlining what is needed to prepare a revision will be listed below. The following reviewer(s) agreed to reveal their identity: Maria Medalla.

The authors have addressed the reviewers concerns.

## Author Response

Reviewer #1

1) The paper deals with quantitative aspects of synapse parameters using samples of

human entorhinal cortex taken postmortem from normal subjects (published earlier)

and AD patients (present data). The authors chose the FIB/SEM technology to pursue

their goal, which is adequate for revealing statistical aspects of synapse morphology

and numerosity. Clearly, the study is heavily statistically oriented in the sense that

neither the origin of the synapses nor that of the postsynaptic structures were

addressed, so that the emerging picture on the form and distribution of synapses in

the neuropil reflects the sheer frequency occurrence without further knowledge of

their position in the neural circuitry. From a technical point of view the measurements

and statistical evaluation have been done correctly with utmost care. However, the

study seems suffering not only from under-sampling (only 4 cases were considered for

each of the two groups, i.e. control and AD) but heterogeneous since one group

contains only female subjects while the other males. Fixing this problem would be

through to obtain controls of the same sex as that of the AD group (which is more

difficult). For example, authors admit that the lower synaptic density found in AD cases

might also be the attributable to the fact that the control group is restricted to male

subjects vs the AD cases were all females. The discrepancy could have been solved if

the control cases have included females, too.

RESPONSE 1

We would like to point out that when dealing with the study of the human brain,

whether it be in normal conditions or in the altered brain such as in Alzheimer’s

disease, the number of cases examined depends on the type of study. For example,

an MRI study on variations in the size of hippocampus is normally based on hundred

or even thousands of cases, because the level of detail and technical constraints

required for such a study makes it possible to use numerous brain samples.

However, if the question is regarding the synaptic organization of the brain, then the

situation is rather different. First, only relatively small samples of the brain can be

examined because the EM techniques are not only time-consuming and laborious

but also require the processing of the brain for EM to occur with a short postmortem

delay (less than 5 h). Thus, it is difficult to obtain “high-quality human brain tissue",

which is why data on human synaptic organization is so scarce. In addition, it is

important to keep in mind that the structure of the human brain is rather variable

compared with experimental animals, which are reared in laboratories under very

homogeneous conditions (breeding, age, environment, etc.). Thus, when studying

the human brain, “n” is practically always 1 unless thousands of brains in different

conditions are examined. We have used 4 control cases and 4 AD, which in our field

of EM is a reasonable number. Of course, if these 8 cases were more homogeneous

in terms of sex and age the study would be more robust, but we are limited to the 2

tissue that is currently available. We really hope to receive more high-quality brain

tissue in the near future. Furthermore, in the discussion, under the subheading

"Interindividual variability", we have dealt with this issue describing the limitations

of this study. Finally, please note that no detailed EM studies have been performed

previously in this particular cortical region of the human brain. Thus, we think that

our study represents an interesting contribution to the field.

To be clearer, we have included the following paragraphs in the Discussion:

"The present data cannot be generalized to the whole population of patients with

AD, which shows clear variability between individuals. Therefore, our study can be

considered as a further step to tackle the issue of the synaptic alterations in

Alzheimer’s disease cases, but it would be necessary to validate our results, both in a

larger number of individuals and in additional brain regions (which we have already

done in the hippocampal CA1 field and in the trans-entorhinal cortex)”.

"Control cases were on average younger (51 years old) than Alzheimer’s disease

cases (85 years old) and it remains uncertain whether some of the synaptic

alterations observed in the present work were due to normal aging or due to the

pathology per se.”

2) All in all the most surprising and unexpected finding is how little, if any, difference is

observed between the control and AD groups regarding synapse morphologies and

some of the quantitative aspects (e.g. no changes in the distribution and types of

postsynaptic targets were found) apart from synapse density values (see paragraph at

line 448). In relation to the observations the really challenging question is spelled out

at line 482, specifically, to decipher cell type related synaptic changes. Since the study

does not answer such a question neither can tell about the functional meaning of the

morphological observation the implications of the synaptic changes is considerably

reduced.

RESPONSE 2

As we have discussed, one of the limitations of the 3D study of the neuropil is that

we cannot identify to which cell type a synapse belongs. However, our sampling

procedure provides us with data from a large number of synapses of a particular

brain region. These synapses can be characterized as asymmetric or symmetric

synapses, which in general correspond to excitatory and inhibitory synapses,

respectively. Therefore, the present data represent the overall balance of

inhibitory/excitatory synapses. This is rather important since it could be the case

that this balance is impaired, as previously shown in other neurological conditions3

such as epilepsy. Deciphering cell type-related synaptic changes is a different subject

that would need to be examined with other methodologies.

3) A major difficulty of this study is to provide a decent conclusion or, how to interpret

the differences in synapse morphologies between control and AD cases. The inherent

limitation in this regard seems to be overcompensated by the exuberant discussion.

For example, the paragraph on functional considerations of synapse takes up 1.5 pages

alone. Some shortening in the overall 10 pages of the discussion would be beneficial to

the paper.

RESPONSE 3

We have shortened the discussion and removed some references.

4) Minor comments:

at line 294, Regarding a 20% decrease in the thickness of EC in AD samples compared

with control cases how could there be a non-significant difference?

RESPONSE

The statistical analysis applied (non-parametrical Mann Whitney U-test for mean

comparison) found that the data were not significantly different. Nevertheless, the

thickness of the EC in AD cases was 20% less than in control cases. This is suggestive

of a real shrinkage of the EC region, although verifying this would require additional

cases.

at line 323, There is a wealth of studies showing that the distribution of synapses is

non-random when the postsynaptic structure is known - i.e. cell morphology, input

type, neurochemical type. For technical reasons, the present work cannot deal with

the above exemplified attributes and possibly explains why the three-dimensional

spatial synaptic distribution is random.

RESPONSE

Of course, there is specificity and selectivity of synaptic contacts established by

cortical neurons, such as -for example- chandelier cells and double bouquet cells.

What we are analyzing here is the general pattern of the distribution of synapses in

the neuropil. It has been described that some pathological structures, like amyloid

plaques and dystrophic dendrites, modify the available space for the synapses’

location (e.g., Blazquez-Llorca et al., 2013 JAD). Thus, considering the AD pathology,

it was important to verify if the 3D synaptic distribution was affected in the plaque-free regions of the brain.4

Reviewer #2

Review for eNeuro (eN-NWR-0504-20): This study is an ultrastructural 3D EM-analysis

of the synaptic and structural differences in layers II and III of the entorhinal cortex

from the brains of control and Alzheimer disease human subjects. The authors

performed stereological methods to assess both cortical atrophy and volume fraction,

and Focused Ion Beam/Scanning Electron Microscopy (FIB/SEM) to examine synaptic

properties. They found a significantly lower volume of neuronal bodies in layer III and a

higher volume of glial cells in layer II in AD affected individuals. AD cases also had

larger and more complex (including horseshoe shaped, perforated or fragmented)

synapses in layer II and a greater proportion of small and simple (macular) synapses in

layer III. While it is recognized that large-scale 3D EM analyses on human brain tissue is

rare, there are some significant concerns and questions pertinent to their analysis,

results and discussion that need to be addressed.

General/Overall:

1) One rather big concern is the balancing of subjects with regards to age and sex. The

discussion adequately describes these caveats with regards to sex differences in AD

phenotypes. However, the bigger concern is aged. Decades worth of normal aging

studies (from Alan Peters, John Morrison) have shown massive synapse loss in normal

aging in prefrontal cortex. The authors need to discuss this and findings for entorhinal

cortex and hippocampal formation (review Morrison and Baxter, 2012).

RESPONSE 1

As we discussed, we did not rule out the effects of age in our study. Nevertheless, in

the discussion, we have added the following paragraph dealing with the previous

studies on aging (see Discussion, line 644):

"In aged rhesus monkey, a lower number of synapses has been reported in

prefrontal cortex related to a cognitive decline; however, studies in rats and

monkeys have shown no evidence of synaptic loss with age in mesial temporal lobe

structures (reviewed in Morrison and Baxter, 2012).”

Moreover, in our previous studies in human temporal lobe (including the

hippocampal CA1 field and transentorhinal cortex), we did not find differences in the

synaptic density in the AD cases compared to control cases. However, light

microscopy studies showed that the thickness of these regions was severely reduced

in AD samples; thus, the results indicate that there is a dramatic loss in the total

number of synapses (Montero-Crespo e al., 2020b; Domínguez-Álvaro et al., 2020).5

2) While a discussion of the caveats can suffice for most cases, the concern about the

magnitude of synapse loss in normal aging, being close to the effect sizes seen

between AD and control in the current study, is a major concern. The differences can

really just be a result of age. If the tissue is available, the authors should add at one or

two control subject close to the age of the AD patients to support the validity of their

conclusions. It is noted that their data for controls were reanalyzed from a previous

work, and adding control cases would certainly strengthen their current study overall.

RESPONSE 2

We agree with the reviewer; if we had more control cases of the same age as the AD

cases, we would have added them to the present study (please, see response 1 to

reviewer 1 above). Unfortunately, we depend on the donation of high-quality brain

tissue from several hospitals, which has been discontinued due to COVID19.

Methods/Results:

1) Given the excellent quality of this dataset, there is some missed opportunity to

describe qualitatively some ultrastructural neuronal abnormalities in AD cases

compared to controls. One particular important question is whether the authors

observe profiles have some abnormal microtubules (“fibrillar tau”) or

endosomes/multivesicular bodies (increased lysosomal/endosomal activity) in

dendrites in some AD cases.

RESPONSE 1

When analyzing the ultrastructural characteristics of the synapses, Alzheimer’s

disease cases had normal-looking synapses that were similar to those observed in

the control cases (see Domínguez-Álvaro et al., 2020). Further evaluation of the

lysosomal/endosomal activity is being performed, and we expect to gather final data

for publication soon.

2) Statistics: The authors should apply a two-way ANOVA for layer and group to see if

there are independent (group and layer) versus interactive (group*layer) effects. Then

when they find main effect ANOVA is significant (for either group, layer or

group*layer), they can report their MW or paired t-tests as valid post-hocs.

RESPONSE 2

We already performed this test, and no interaction was found; only differences

between groups (AD versus Control) were found. The results presented in the paper

show the statistically significant differences found by the non-parametrical U-Mann

Whitney test.6

3) For the first few Results sections showing Cortical Thickness, and volume fraction

analyses, it is unclear in the Results what tissue/analyses was used. While this is

described in the Methods, it would help if the authors clarify in the Results sections if

analyses were derived from light microscope immune-stained sections or semi-thin

toludine blue (serial sections)

RESPONSE 3

We have modified the Results section as follows:

"Measurements of the cortical thickness were derived from semi-thin toludine blue

(serial sections) using light microscopy.”

4) Cortical thickness: It is unclear how the authors measured this. IT states in the

methods that they measure this on vibratome sections. Did they measure from

multiple points of the pia across the entire EC? Then run a perpendicular line from

each point to the beginning of the white matter?

RESPONSE 4

To address this point, in the Methods section we have changed this section and it

now reads as follows:

"In order to estimate the atrophy of the EC, we measured the cortical thickness in

three to five toluidine blue-stained semithin sections from each of the cases,

obtained in the coronal plane of the cortex and containing the entire cortex, tracing a

perpendicular line from the pial surface to the white matter. Measurements of the

distance between the pial surface and the boundary with the white matter were

performed using Fiji program (ImageJ 1.51; NIH, USA; http://imagej.nih.gov/ij/).To

average data, three measurements were made per section.”

5) Cortical thickness: Results section should site Figure 1

RESPONSE 5

This is now corrected.

6) Lines 297-298: In the control group layer II, the values of the estimated volume

fraction (Vv) occupied by neuronal somata, glial somata

As noted above, it would be helpful if it was clarified that these volume fraction

estimates were from (serial?) semithin sections (1-2μm thick) stained with 1%

toluidine. Further in Figure 5, it would be nice to see an example of the segmentation

on the actual tissue to show how the quantification was done.7

RESPONSE 6

This issue has been dealt with above in responses 3 and 4.

7) Lines 323-321 Three-dimensional spatial synaptic distribution: “Analysis indicates a

clear fit to a CSR model, since F, G and K functions closely resemble the theoretical

curve that these functions represent, both in the control and the AD cases.”

Unclear what this statement is referring too. Please define CSR here, and elaborate on

the rationale of this analyses, include a citation or even a figure to illustrate this

analysis.

RESPONSE 7

We have changed this section and it now reads as follows:

"Analysis indicates a clear fit to a complete spatial randomness (CSR) model, since F,

G and K functions closely resemble the theoretical curve that these functions

represent, both in the control and the AD cases. The CSR model defines a situation

where a point is equally likely to occur at any location within the study volume,

regardless of the locations of other points. Therefore, a CSR model is considered as a

reference for a random pattern in spatial point process statistics (reviewed in

Merchán-Pérez et al., 2014).”

8) The numbers of SS were very low, and perhaps can be due to some of these

methodological constraints of identifying these in FIB/SEM? How do these compare to

previous estimates in EC humans/monkeys?

RESPONSE 8

It is true that the proportion of SS is very low. However, hundreds of synapses were

identified in the images stacks. As described in the Material and Methods section:

"Since the EspINA software allows navigation through the stack of images (Figs. 3, 4), it

was possible to unambiguously identify every synapse as an asymmetric synapse (AS)

or a symmetric synapse (SS), based on the thickness of the PSD (Fig. 4)"

Additionally, we have compared our results with previous estimations, and have

added this paragraph to the discussion:

"Previous studies have shown that the percentage of AS and SS varies -80-95% and

20-5%, respectively- in all the cortical layers, cortical areas and species examined

so far by TEM (Beaulieu and Colonnier, 1985; Megıás et al., 2001; Bourne and Harris,

2011; DeFelipe 2011, 2015). Estimations with samples from human brain using the

same method have shown that the AS:SS ratio in layer III of the temporal area 21 is8

93:7 (Cano-Astorga et al., 2021), 96:4 in the transentorhinal cortex (Dominguez-Alvaro et al., 2018), and 95:5 in the human CA1 hippocampal field (except in the

stratum lacunosum moleculare, in which this ratio was 89:11; Montero-Crespo et al.,

2020). Thus, it would appear that the present AS:SS ratio data provide among the

highest and lowest proportions previously observed in different brain regions, for AS

and SS, respectively.”

Figures and Legends

9) Figure 2-3 should be combined as they represent more methods.

RESPONSE 9

We can see the reviewer's point but think it is preferable to keep them separate so

that they can be printed in larger dimensions.

10) Figure 1 top panel should be labeled as panel A and should have its own scale bar

and axes (M-L, D-V).

RESPONSE 10

Figure 1 plots now have their own scale bar as suggested by the reviewer.

11) Figure 4: Please also label the type of postsynaptic target in EM image (AS on spine,

SS on dendrite). Adding more micrographs to show examples of all synapse

types/targets identified.

RESPONSE 11

Figure 4 has been relabeled as recommended.

We have included a new figure (Fig. 5) of a dendritic shaft segment fully 3D

reconstructed showing diverse postsynaptic targets and their corresponding

synapses. Additionally, two movies of the 3D reconstructed dendrite have been

prepared to illustrate examples of these postsynaptic targets.

12) Figure 8 legend text is truncated.

RESPONSE 12

This problem has been fixed.

13) Minor concern: I am unclear about the organization of the “extended

data/figures”. I am not sure why some “Tables” in the extended data are labeled as

"Fig", specifically:

RESPONSE 139

We are sorry, we probably misunderstood e-Neuro instructions, because the

extended tables/figures must be labeled as related to an original table/figure. We

have re-labeled them.

Table Fig. 5-1. Volume fraction occupied by cortical elements in layers II and III of the

EC

RESPONSE

This has now been corrected.

Table Fig. 5-2. Light microscopy data on volume fraction occupied by cortical elements

in layers II and III of the EC for individual cases.

RESPONSE

This has now been corrected.

Fig. 8-1. Area (nm ) and perimeter (nm) of the macular, perforated, horseshoe shaped

and fragmented SAS in layer II of the EC.

RESPONSE

This has now been corrected.

Fig. 8-2. Area (nm2 ) and perimeter (nm) of the macular, perforated, horseshoe

shaped and fragmented SAS in layer III of the EC.

RESPONSE

This has now been corrected.

Discussion:

14) Lines 392-398: First paragraph of the Discussion should specify/state that the data

is from human Entorhinal Cortex.

RESPONSE

It is now stated that the data are from human Entorhinal Cortex.

15) lines 498-500: “Therefore, the random distribution pattern of synapses in the

neuropil does not appear to be affected by the lower synaptic density in AD cases.”

Please elaborate more in the functional consequence of this finding/statement. It

seems out of place after the previous section describing the highly specific cell-type

and compartmental distribution of excitatory and inhibitory synapses.

RESPONSE 15

We have now included the following paragraphs:10

"The present results indicate that the distribution of synapses in the neuropil of both

control and AD samples is nearly random, only constrained by the fact that synapses

cannot physically overlap in space and so their geometric centers or centroids cannot

be too close to their neighbors. As we have previously shown in human brain

samples from hippocampal CA1, entorhinal cortex, transentorhinal cortex and

temporal cortex, there seems to be no limitation to the position of any synapse

except the space already occupied by other synapses (Montero-Crespo et al., 2020;

Dominguez-Alvaro et al., 2018, 2021; Cano-Astorga et al., 2021).”

"It has been reported that during cortical development, synapses are randomly

added and/or withdrawn from the population, as described by the synaptic turnover

(Rakic et al. 1994). It seems that the only constraint for a synapse to form is that this

particular spot is not already occupied by a preexisting synapse (reviewed in

Merchán-Pérez et al., 2014). Interestingly, in the AD cases, the loss of synapses per

unit volume does not affect their 3D distribution.”

16) Lines 512-522: The following statement has some redundancies: The parenthetical

in Lines 517-520 “..... probability of neurotransmitter release suggest that larger

synapses (with a greater number of postsynaptic receptors, mainly AMPA receptors)

may produce more powerful, homogeneous and numerous synaptic responses, while

smaller synapses (with fewer receptors, mainly NMDA) may produce weaker and more

variable responses.... “ is redundant with the following sentence (lines 521-522). “Also,

larger synapses have a higher number of AMPA receptors, while smaller synapses have

a higher concentration of NMDA receptors (Kharazia and Weinberg, 1999).

RESPONSE 16

The discussion has been reviewed to avoid redundancies.

17) Lines 533-535: “Under normal conditions, macular synapses have the capacity to

progressively increase their perimeter, adopting a more tortuous perimeter with

perforations and indentations (Geinisman et al., 1987; 1991; 1992a, b).” Statement is

unclear, please elaborate and clarify. I presume that the authors are referring to these

studies showing the dynamic nature of synapses transforming from macular to

perforated to eventually splitting into two synapses? Perhaps state how “macular

synapses are thought to have the capacity to change morphologies and develop

perforations and indentations in an activity-dependent manner...”

I am also unclear how this statement fits with the logic of the previous and following

statement/discussions.

RESPONSE 17

This section of the discussion has been edited as suggested by the reviewer.11

18) Lines 561-562: “it could be 561 hypothesized that the morphological changes in AD

would trigger compensatory mechanisms during the development of the disease.”

Perhaps using the word “progression” instead of “development” is more appropriate.

RESPONSE 18

This has now been corrected.

19) In the statement (lines 575-577): “In addition, since the majority of the synapses

that a pyramidal cell receives are on spines and they represent the vast majority of AS

synapses (DeFelipe and Fariñas,1992), the present data do not seem to be related to a

loss of spines.”

The statement “the present data do not seem to be related to a loss of spines.” Is not

consistent with the 40% decrease in density of asymmetric synapses (most of which

are on spines). Their data is indeed consistent with a loss of spines. Their data on

decreased neuronal fraction in layer II of EC also suggest that this spine loss is likely

due to the lower neuron number in layer III of EC, instead of dendritic or spine

regression. To answer this definitively, the authors can do 3D analyses on individual

dendrites and count spines coming off each one of them (a challenging analysis) or

quantify total dendritic length in their volume analyzed and divide this by the total

axospinous asymmetric synapses to estimate spines/dendritic length.

RESPONSE 19

It is true that the majority of the synapses that a pyramidal cell receives are on

spines and, in general, they represent the vast majority of AS synapses. We

completely agree with the reviewer that 3D analyses of individual dendrites would

provide further information and would help to understand the human connectome

and its possible alterations under pathological conditions. However, performing this

study with thousands of synapses, dendrites and axons would be extremely time-consuming and technically challenging. Certainly, we could carry out this study with

a relatively small subset of the reconstructed synapses to accelerate the process, but

the obtained connectivity data would be less robust than if we used all of the

synapses that we were able to reconstruct. For these reasons, our laboratory is

currently developing a software tool that will make it possible to accurately and

reliably trace and reconstruct a large number of axonal and dendritic segments

contained within the FIB/SEM stacks.

In our opinion, this analysis would make it possible to accurately answer important

questions regarding pre- and post-synaptic elements in detail and in relation to their

parental neuronal processes. Thus, we are expecting to answer these questions in

the near future and, respectfully, we would like to leave the answer to this question

for a future study.12

Nevertheless, to improve the accuracy of our postsynaptic target data, all the

FIB/SEM stacks -from both control and Alzheimer’s disease samples- have been

re-examined and classified. The new data have been added to the text and tables.

Accordingly, we have modified the Material & Methods section as follows:

"Unambiguous identification of spines as postsynaptic targets requires the spine to

be visually traced to the parent dendrite, yielding what we refer to as fully

reconstructed spines. Additionally, when synapses were established on a spine head-shaped postsynaptic element whose neck could not be followed to the parent

dendrite, or whose neck was truncated, we identified these elements as non-fully

reconstructed spines. These non-fully reconstructed spines were identified on the

basis of their size and shape, the lack of mitochondria and the presence of a spine

apparatus - or because they were filled with a characteristic fluffy material (used to

describe the fine and indistinct filaments present in the spines) - a term coined by

Peters et al. (1991) (see also del Río and DeFelipe, 1995). For simplicity, we will refer

to the fully reconstructed and non-fully reconstructed spines as spines, unless

otherwise specified.”

The inclusion of these data attempts to provide more accurate results regarding the

proportion of the postsynaptic targets. These new data have been added to tables 5,

5-1 and 5-2, as well as in the results section.

According to the reviewer’s comment, we have modified the discussion, as follows:

"In addition, the majority of the synapses that a pyramidal cell receives are on spines

and they represent the vast majority of AS synapses (DeFelipe and Fariñas, 1992). In

the human transentorhinal cortex, 75% of the synapses were AS on spines, which is

similar to the case of the AS found in the layer III of the temporal cortex (75%) using

the same analysis method (Cano-Astorga et al., 2021). This percentage was much

higher in the CA1 hippocampal field, as high as 94% in the superficial part of the CA1

stratum pyramidale (Montero-Crespo et al., 2020).

In both layers II and III of the entorhinal cortex, the percentage of AS on spines was

57%; 43% in the case of AS on dendritic shafts. This is a higher proportion of AS on

shafts than in the other human cortical regions previously analyzed using the same

techniques.

In addition, the comparison between control and Alzheimer’s disease samples did

not show a clear reduction in the proportion of AS on spines. Only a slightly lower

proportion of AS established on spines was found in layer II from Alzheimer’s disease

samples. Therefore, the lower synaptic density does not seem to be specifically

related to a loss of spines, but rather a general loss of synapses might occur

regardless of their postsynaptic targets.”13

20) Lines 584-586: EC provides excitatory inputs to the hippocampus: the indirect

pathway (trisynaptic), from layer II neurons, and the direct pathway (monosynaptic)

from layer III (reviewed in Insausti and Amaral, 2012; Marks et al., 2020).

This statement is confusing, both the tri-synaptic and monosynaptic perforant

pathways are directly targeting the hippocampus albeit different parts (dentate gyrus

versus CA1 directly). Did the authors mean to say inputs to CA1 instead of

"hippocampus”? Or just remove the words indirect or direct to read something like:

"provides excitatory inputs to the hippocampus from layer II neurons targeting the

dentate gyrus (trisynaptic pathway) and from layer III neurons monosynaptically

targeting CA1 (reviewed in Insausti and Amaral, 2012; Marks et al., 2020).”

RESPONSE 20

Thank you for this comment. We have changed this paragraph as suggested by the

reviewer. It now reads as follows:

"EC provides excitatory inputs to the hippocampus from layer II neurons targeting

the dentate gyrus (trisynaptic pathway) and from layer III neurons monosynaptically

targeting CA1 (reviewed in Insausti and Amaral, 2012; Marks et al., 2020).”

21) Lines 597-598: Layer III receives excitatory inputs from these same regions, but

also receives inputs from the claustrum and the amygdala (reviewed in Insausti and

Amaral, 2012; Schultz et al., 2015). Best to specify in monkeys (including species) when

referring to connectivity. Also, some work from the Barbas lab for the amygdala to

entorhinal connections can be added here. Also, there are inputs from the medial and

orbital prefrontal cortex that maybe worth mentioning (Work from the Barbas

[reviewed in {Barbas 2013}] and Price groups).

RESPONSE 21

To be clearer and shorten the discussion, as suggested by the reviewer, we have

changed this paragraph; it now reads as follows:

"Moreover, in layer II there are two subpopulations of excitatory neurons: spiny

stellate neurons (which project to DG, CA3 and CA2) and modified pyramidal neurons

(which project to stratum lacunosum in CA1). Projections from these neurons,

constituting the trisynaptic pathway, seem to be related to contextual memories

(reviewed in Mark et al., 2020). It has been proposed that this pathway is more

susceptible to premature degeneration than the monosynaptic pathway (Van

Hoesen et al., 2006; Llorens-Martín et al., 2014).

In addition, pyramidal neurons from layer III directly project to pyramidal neuron

dendrites located in the stratum moleculare of CA1, and it has been proposed that 14

these monosynaptic excitatory inputs drive temporal association learning (reviewed

in Mark et al., 2020).”

22) Lines 478-482: However, it has been shown that there are differences in the

number of GABAergic and glutamatergic synaptic inputs in different neuronal types in

other cortical regions of a variety of species (e.g., DeFelipe and Fariñas, 1992; Freund

and Buzsáki, 1996; DeFelipe, 1997; Somogyi et al., 1998; Schubert et al., 2007;

Markram et al., 2015;Tremblay et al., 2016).

Comparative studies from the Medalla/Luebke group have also shown some

differences in 3D ultrastructural features by area and species. Perhaps worth

mentioning here too (Hsu et al. 2017).

RESPONSE 22

This paragraph has been changed to:

"However, it has been shown that there are differences in the number of GABAergic

and glutamatergic synaptic inputs in different neuronal types in other cortical

regions of a variety of species (e.g., DeFelipe and Fariñas, 1992; Freund and Buzsáki,

1996; DeFelipe, 1997; Somogyi et al., 1998; Schubert et al., 2007; Markram et al.,

2015; Tremblay et al., 2016; Hsu et al., 2017).”

Minor points and typos:

23) 376 element was analyzed - space is missing

24) Lines 401-402: “the difference in the mean cortical thickness was no evident (Table

1-1)” - should be “not evident"

25) Lines 415-418:Since our present neuronal volume fraction estimations are based

on semithin sections, and different types of neurons have not been identified, we

cannot rule out a selective neuronal loss that may affect either “to excitatory neurons

or to GABAergic interneurons or to both cell types.” Seems like grammatical use of “to"

is incorrect here, and the sentence is unclear. Are the authors referring to the loss

affecting (selective loss of) either excitatory, inhibitory or both? If so, delete the word

"to"

RESPONSE

The manuscript has been revised and edited according to the reviewer’s comments.

We would like to thank the reviewer for drawing our attention to these points
