# Supplementary material for: 3D Analysis of the Synaptic Organization in the Entorhinal Cortex in Alzheimer’s Disease
Source: eNeuro. 2021 Jun 18;8(3):ENEURO.0504-20.2021. doi: 10.1523/ENEURO.0504-20.2021 (PMC8225407; doi:10.1523/ENEURO.0504-20.2021)
Supplement: Extended Data Table 2-1 — Data from the ultrastructural analysis of neuropil from Layers II and III of the EC for individual cases. All volume and distance data are corrected for shrinkage. All, includes AS+SS synapses; No., number. Download Table 2-1, DOCX file. [file enu-eN-NWR-0504-20-s01.docx]

**Extended Table 1-1. EC cortical thickness in control and Alzheimer cases.**

| **Group** | **Case** | **EC thickness**  **(mm; mean± SD)** |
| --- | --- | --- |
| **Control** | **AB1** | 2.12±0.06 |
|  | **AB3** | 2.08±0.01 |
|  | **IF10** | 1.75±0.05 |
|  | **M16** | 2.04±0.02 |
| **Alzheimer** | **IF1** | 2.30±0.03 |
|  | **VK11** | 1.40±0.01 |
|  | **VK16** | 1.19±0.01 |
|  | **VK22** | 1.51±0.02 |

**Extended Table 1-2. Volume fraction occupied by cortical elements in layers II and III of the EC.**

| **EC layer** | **Group** | **V_neu_ (%)** | **V_g_ (%)** | **V_bv_ (%)** | **V_n_ (%)** |
| --- | --- | --- | --- | --- | --- |
| **II** | **Control** | 4.49±2.91 | 2.64±1.05 | 3.80±2.31 | 89.07±3.67 |
|  | **Alzheimer** | 2.61±1.41 | 4.59±0.90 | 2.34±1.59 | 90.47±2.03 |
| **II** | **Control** | 7.53±1.78 | 2.52±0.42 | 3.80±0.74 | 86.15±1.23 |
|  | **Alzheimer** | 2.88±1.48 | 4.55±1.85 | 3.98±2.73 | 88.59±3.94 |

**Extended Table 1-3. Light microscopy data on volume fraction occupied by cortical elements in layers II and III of the EC for individual cases.**

| **EC layer** | **Group** | **Case** | **V_neu_**  **(%; mean)** | **V_g_**  **(%; mean)** | **V_vs_**  **(%; mean)** | **V_n_**  **(%; mean)** |
| --- | --- | --- | --- | --- | --- | --- |
| **II** | **Control** | **AB1** | 4.19 | 1.74 | 2.56 | 91.50 |
|  |  | **AB3** | 8.67 | 2.44 | 2.72 | 86.17 |
|  |  | **IF10** | 2.86 | 4.16 | 7.29 | 85.69 |
|  |  | **M16** | 2.23 | 2.23 | 2.62 | 92.91 |
|  | **Alzheimer** | **IF1** | 3.97 | 3.44 | 3.84 | 88.75 |
|  |  | **VK11** | 2.88 | 4.68 | 3.55 | 88.89 |
|  |  | **VK16** | 0.63 | 5.65 | 0.75 | 92.97 |
|  |  | **VK22** | 2.96 | 4.59 | 1.20 | 91.25 |
| **III** | **Control** | **AB1** | 9.56 | 2.23 | 3.82 | 84.39 |
|  |  | **AB3** | 7.82 | 2.10 | 3.11 | 86.97 |
|  |  | **IF10** | 5.22 | 2.95 | 4.82 | 87.01 |
|  |  | **M16** | 7.53 | 2.80 | 3.44 | 86.23 |
|  | **Alzheimer** | **IF1** | 4.29 | 2.40 | 3.44 | 89.87 |
|  |  | **VK11** | 3.99 | 4.66 | 5.69 | 85.66 |
|  |  | **VK16** | 1.79 | 4.24 | 0.36 | 93.60 |
|  |  | **VK22** | 1.41 | 6.91 | 6.44 | 85.24 |

**Extended Table 2-1. Data from the ultrastructural analysis of neuropil from layers II and III of the EC for individual cases.**

| **EC layer** | **Case** | **No. AS**  **synapses** | **No. SS**  **synapses** | **No. all**  **synapses** | **% AS**  **Synapses**  **(mean ± SD)** | **% SS**  **Synapses**  **(mean ± SD)** | **CF**  **volume**  **(μm^3^)** | **No. AS**  **synapses/μm^3^**  **(mean ± SD)** | **No. AS**  **synapses/μm^3^**  **(mean ± SD)** | **No. synapses/μm^3^**  **(mean ± SD)** | **Distance to nearest neighbor**  **(nm; mean)** |
| --- | --- | --- | --- | --- | --- | --- | --- | --- | --- | --- | --- |
| **II** | **AB1** | 379 | 27 | 406 | 93.11±2.86 | 6.89±2.86 | 986 | 0.39±0.07 | 0.02±0.01 | 0.41±0.06 | 848 |
|  | **AB3** | 397 | 39 | 436 | 90.81±1.10 | 9.19±1.10 | 1139 | 0.35±0.09 | 0.03±0.00 | 0.38±0.09 | 883 |
|  | **IF10** | 376 | 22 | 398 | 94.50±1.43 | 5.50±1.43 | 1059 | 0.36±0.04 | 0.02±0.01 | 0.38±0.04 | 801 |
|  | **M16** | 401 | 49 | 450 | 89.18±1.06 | 10.82±1.06 | 1037 | 0.39±0.01 | 0.04±0.00 | 0.43±0.00 | 828 |
|  | **IF1** | 219 | 20 | 239 | 91.50±1.65 | 8.50±1.65 | 1128 | 0.19±0.02 | 0.02±0.00 | 0.21±0.02 | 1021 |
|  | **VK11** | 313 | 17 | 330 | 95.55±2.55 | 4.45±2.55 | 998 | 0.30±0.07 | 0.02±0.01 | 0.32±0.08 | 824 |
|  | **VK16** | 126 | 22 | 148 | 85.23±1.51 | 14.77±1.51 | 1186 | 0.10±0.01 | 0.02±0.00 | 0.12±0.02 | 1049 |
|  | **VK22** | 296 | 31 | 327 | 89.86±3.67 | 10.14±3.67 | 1097 | 0.26±0.05 | 0.03±0.01 | 0.29±0.04 | 860 |
| **III** | **AB1** | 421 | 36 | 457 | 92.12±1.15 | 7.88±1.15 | 1049 | 0.40±0.03 | 0.03±0.01 | 0.44±0.04 | 849 |
|  | **AB3** | 446 | 35 | 481 | 92.84±0.92 | 7.16±0.92 | 1089 | 0.41±0.04 | 0.03±0.01 | 0.44±0.04 | 903 |
|  | **IF10** | 469 | 27 | 496 | 94.14±1.53 | 5.86±1.53 | 1143 | 0.41±0.17 | 0.02±0.00 | 0.43±0.17 | 799 |
|  | **M16** | 411 | 32 | 443 | 92.27±3.61 | 7.73±3.61 | 1090 | 0.38±0.11 | 0.03±0.01 | 0.41±0.11 | 857 |
|  | **IF1** | 298 | 40 | 338 | 87.08±11.64 | 12.92±11.64 | 1240 | 0.24±0.07 | 0.03±0.02 | 0.27±0.06 | 951 |
|  | **VK11** | 265 | 13 | 278 | 95.36±0.56 | 4.64±0.56 | 1040 | 0.26±0.06 | 0.01±0.00 | 0.27±0.06 | 869 |
|  | **VK16** | 152 | 2 | 154 | 98.50±1.85 | 1.50±1.85 | 1238 | 0.12±0.08 | 0.00±0.00 | 0.12±0.08 | 1320 |
|  | **VK22** | 258 | 37 | 295 | 87.55±1.09 | 12.45±1.09 | 1079 | 0.24±0.04 | 0.03±0.01 | 0.27±0.05 | 877 |

**Extended Table 3-1. Area (nm^2^) and perimeter (nm) of the SAS in layers II and III of the EC** **for individual cases.**

|  |  | **Layer II** | | **Layer III** | |
| --- | --- | --- | --- | --- | --- |
| **Case** | **Type of synapse** | **Area of SAS** | **Perimeter of SAS** | **Area of SAS** | **Perimeter of SAS** |
| **AB1** | AS | 101,766±3757 | 1551±40 | 116,843±3,974 | 1673±43 |
|  | SS | 58,790±6865 | 1252±117 | 60,978±5,015 | 1220±79 |
| **AB3** | AS | 109,320±3694 | 1605±38 | 132,252±4,051 | 1859±47 |
|  | SS | 71,082±13,154 | 1593±226 | 67,083±5,083 | 1444±89 |
| **IF10** | AS | 113,399±4343 | 1770±51 | 121,736±4,345 | 1991±60 |
|  | SS | 53,681±4642 | 1331±112 | 65,338±9,444 | 1578±210 |
| **M16** | AS | 116,757±4139 | 1598±40 | 125,900±4,790 | 1779±55 |
|  | SS | 80,435±6621 | 1440±73 | 76,379±10,422 | 1354±129 |
| **IF1** | AS | 163,933±7143 | 1909±54 | 146,264±5,934 | 1975±72 |
|  | SS | 77,503±12,221 | 1422±141 | 99,073±8,446 | 2149±170 |
| **VK11** | AS | 120,779±5614 | 1858±66 | 134,348±7,330 | 1858±83 |
|  | SS | 53,824±6035 | 1236±80 | 46,814±13,543 | 1161±254 |
| **VK16** | AS | 142,393±15,315 | 1841±131 | 106,568±7,258 | 1511±53 |
|  | SS | 46,667±5245 | 1070±77 | 46,515±7,167 | 1103±20 |
| **VK22** | AS | 117,558±5198 | 1668±50 | 103,726±5,248 | 1536±52 |
|  | SS | 58,932±6912 | 1227±87 | 36,680±2,946 | 959±56 |

**Extended Table 4-1. Proportion of macular, perforated, horseshoe-shaped and fragmented synapses in layer II of the EC for individual cases.**

| **Case** | **Type of synapse** | **Macular**  **synapses** | **Perforated synapses** | **Horseshoe synapses** | **Fragmented synapses** | **Total synapses** |
| --- | --- | --- | --- | --- | --- | --- |
| **AB1** | AS | 84.7% (320) | 9.8% (37) | 4.5% (17) | 1.0% (4) | 100% (378) |
|  | SS | 74.1% (20) | 7.4% (2) | 18.5% (5) | 0% (0) | 100% (27) |
| **AB3** | AS | 82.6% (327) | 13.6% (54) | 2.5% (10) | 1.3% (5) | 100% (396) |
|  | SS | 66.6% (26) | 15.4% (6) | 15.4% (6) | 2.6% (1) | 100% (39) |
| **IF10** | AS | 81.6% (307) | 14.1% (53) | 4.0% (15) | 0.3% (1) | 100% (376) |
|  | SS | 72.7% (16) | 9.1% (2) | 18.2% (4) | 0% (0) | 100% (22) |
| **M16** | AS | 83.0% (331) | 14.0% (56) | 2.3% (9) | 0.7% (3) | 100% (399) |
|  | SS | 77.6% (38) | 10.2% (5) | 12.2% (6) | 0% (0) | 100% (49) |
| **IF1** | AS | 86.8% (190) | 9.6% (21) | 3.2% (7) | 0.4% (1) | 100% (219) |
|  | SS | 70.0% (14) | 15.0% (3) | 15.0% (3) | 0% (0) | 100% (20) |
| **VK11** | AS | 77.3% (242) | 14.7% (46) | 6.1% (19) | 1.9% (6) | 100% (313) |
|  | SS | 58.8% (10) | 23.5% (4) | 11.8% (2) | 5.9% (1) | 100% (17) |
| **VK16** | AS | 79.4% (100) | 19.0% (24) | 1.6% (2) | 0% (0) | 100% (126) |
|  | SS | 77.3% (17) | 0% (0) | 18.2% (4) | 4.5% (1) | 100% (22) |
| **VK22** | AS | 80.1% (237) | 12.5% (37) | 6.4% (19) | 1.0% (3) | 100% (296) |
|  | SS | 80.6% (25) | 12.9% (4) | 6.5% (2) | 0% (0) | 100% (31) |

**Extended Table 4-2. Proportion of macular, perforated, horseshoe-shaped and fragmented synapses in layer III of the EC for individual cases.**

| **Case** | **Type of synapse** | **Macular**  **synapses** | **Perforated synapses** | **Horseshoe synapses** | **Fragmented synapses** | **Total synapses** |
| --- | --- | --- | --- | --- | --- | --- |
| **AB1** | AS | 78.6% (330) | 17.6% (74) | 3.6% (15) | 0.2% (1) | 100% (420) |
|  | SS | 80.0% (28) | 5.7% (2) | 11.4% (4) | 2.9% (1) | 100% (35) |
| **AB3** | AS | 73.5% (328) | 21.1% (94) | 3.4% (15) | 2.0% (9) | 100% (446) |
|  | SS | 48.6% (17) | 14.3% (5) | 37.1% (13) | 0% (0) | 100% (35) |
| **IF10** | AS | 76.3% (358) | 19.6% (92) | 3.7% (17) | 0.4% (2) | 100% (469) |
|  | SS | 66.7% (18) | 22.2% (6) | 11.1% (3) | 0% (0) | 100% (27) |
| **M16** | AS | 75.4% (310) | 21.2% (87) | 2.7% (11) | 0.7% (3) | 100% (411) |
|  | SS | 68.8% (22) | 21.9% (7) | 6.2% (2) | 3.1% (1) | 100% (32) |
| **IF1** | AS | 78.5% (234) | 10.1% (30) | 6.7% (20) | 4.7% (14) | 100% (298) |
|  | SS | 60.0% (24) | 12.5% (5) | 17.5% (7) | 10.0% (4) | 100% (40) |
| **VK11** | AS | 77.4% (205) | 15.8% (42) | 3.8% (10) | 3.0% (8) | 100% (265) |
|  | SS | 76.9% (10) | 7.7% (1) | 7.7% (1) | 7.7% (1) | 100% (13) |
| **VK16** | AS | 92.8% (141) | 4.6% (7) | 2.6% (4) | 0% (0) | 100% (152) |
|  | SS | 100.0% (2) | 0% (0) | 0% (0) | 0% (0) | 100% (2) |
| **VK22** | AS | 87.6% (226) | 7.7% (20) | 3.1% (8) | 1.6% (4) | 100% (258) |
|  | SS | 86.5% (32) | 5.4% (2) | 5.4% (2) | 2.7% (1) | 100% (37) |

**Extended Table 4-3. Area (nm^2^) and perimeter (nm) of the macular, perforated, horseshoe-shaped and fragmented SAS in layer II of the EC.**

| **Group** | **Shape** | **Type of synapse** | **SAS Area**  **(nm^2^; mean± sem)** | **SAS Perimeter (nm; mean± sem)** |
| --- | --- | --- | --- | --- |
| **Control** | **Macular** | AS | 89,406±1999 | 1389±21 |
|  |  | SS | 53,716±7280 | 1126±32 |
|  | **Perforated** | AS | 218,803±7373 | 2698±121 |
|  |  | SS | 104,339±22,261 | 1730±413 |
|  | **Horseshoe** | AS | 191,238±14,403 | 3242±273 |
|  |  | SS | 84,034±6931 | 2046±47 |
|  | **Fragmented** | AS | 199,121±19,630 | 2818±269 |
|  |  | SS | 152,108±0 | 2500±0 |
| **Alzheimer** | **Macular** | AS | 101,410±13,147 | 1474±87 |
|  |  | SS | 50,519±4291 | 1101±59 |
|  | **Perforated** | AS | 290,075±33,353 | 3146±261 |
|  |  | SS | 87,027±14,137 | 1584±95 |
|  | **Horseshoe** | AS | 230,872±27,294 | 3296±196 |
|  |  | SS | 95,635±24,799 | 1821±223 |
|  | **Fragmented** | AS | 329,742±94,617 | 3993±782 |
|  |  | SS | 54,638±11,867 | 1323±150 |

**Extended Table 4-4. Area (nm^2^) and perimeter (nm) of the macular, perforated, horseshoe-shaped and fragmented SAS in layer III of the EC.**

| **Group** | **Shape** | **Type of synapse** | **SAS Area**  **(nm^2^; mean± sem)** | **SAS Perimeter (nm; mean± sem)** |
| --- | --- | --- | --- | --- |
| **Control** | **Macular** | AS | 92,737±3846 | 1430±32.73 |
|  |  | SS | 65,303±14,798 | 1110±27.5 |
|  | **Perforated** | AS | 228,057±6993 | 2964±173.7 |
|  |  | SS | 86,820±5148 | 1695±86.54 |
|  | **Horseshoe** | AS | 18,8362±9007 | 3447±340.4 |
|  |  | SS | 157,439±53,309 | 2343±588.2 |
|  | **Fragmented** | AS | 303,754±35,891 | 4254±505.9 |
|  |  | SS | 62,518±5339 | 2583±1624 |
| **Alzheimer** | **Macular** | AS | 96,162±6492 | 1412±47.63 |
|  |  | SS | 47,435±9453 | 1120±156.6 |
|  | **Perforated** | AS | 296,156±24,116 | 3192±150.9 |
|  |  | SS | 99,845±47,349 | 2723±868.4 |
|  | **Horseshoe** | AS | 192,661±19,980 | 3117±283.5 |
|  |  | SS | 127,306±39,972 | 2289±479.0 |
|  | **Fragmented** | AS | 250,707±40,241 | 3577±781.0 |
|  |  | SS | 70,021±43,790 | 1758±908.2 |

**Extended Table 5-1. Distribution of AS and SS on spines and dendritic shafts in layer II of the EC for individual cases.**

| **Case** | **Type of synapse** | **Synapses on spine heads** | **Synapses on nf, spine heads** | **Synapses on spine necks** | **Synapses on aspiny dendritic shafts** | **Synapses on spiny dendritic shafts** | **Total synapses** |
| --- | --- | --- | --- | --- | --- | --- | --- |
| AB1 | AS | 35.3% (121) | 20.7% (71) | 0.6% (2) | 26.5% (91) | 16.9% (58) | 100% (343) |
|  | SS | 4.8% (1) | 4.8% (1) | 0.0% (0) | 33.3% (7) | 57.1% (12) | 100% (21) |
| AB3 | AS | 31.6% (116) | 17.9% (66) | 0.6% (2) | 24.8 (91) | 25.1% (92) | 100% (367) |
|  | SS | 10.8% (4) | 5.4% (2) | 0.0% (0) | 27.0% (10) | 56.8% (21) | 100% (37) |
| IF10 | AS | 36.7% (122) | 22.6% (75) | 0.3% (1) | 17.2% (57) | 23.2% (77) | 100% (332) |
|  | SS | 9.1% (2) | 9.1% (2) | 0.0% (0) | 18.2% (4) | 63.6% (14) | 100% (22) |
| M16 | AS | 53.7% (208) | 15.0% (58) | 0.8% (3) | 11.6% (45) | 18.9% (73) | 100% (387) |
|  | SS | 10.4 (5) | 2.1% (1) | 2.1% (1) | 16.7% (8) | 68.7% (33) | 100% (48) |
| IF1 | AS | 34.4% (71) | 15.9% (33) | 0.5% (1) | 21.7% (45) | 27.5% (57) | 100%( 207) |
|  | SS | 20.0% (4) | 5.0% (1) | 0.0% (0) | 35.0% (7) | 40.0% (8) | 100% (20) |
| VK11 | AS | 35.5% (89) | 23.5% (59) | 0.8% (2) | 20.7% (52) | 19.5% (49) | 100% (251) |
|  | SS | 13.3% (2) | 0.0% (0) | 0.0% (0) | 40.0% (6) | 46.7% (7) | 100% (15) |
| VK16 | AS | 27.1% (26) | 21.9% (21) | 3.1% (3) | 32.3% (31) | 15.6% (15) | 100% (96) |
|  | SS | 5.3% (1) | 0.0% (0) | 0.0% (0) | 57.9% (11) | 36.8% (7) | 100% (19) |
| VK22 | AS | 38.7% (98) | 12.3% (31) | 0.4% (1) | 29.6% (75) | 19.0% (48) | 100% (253) |
|  | SS | 7.1% (2) | 0.0% (0) | 0.0% (0) | 57.2% (16) | 35.7% (10) | 100% (28) |

**Extended Table 5-2. Distribution of AS and SS on spines and dendritic shafts in layer III of the EC for individual cases.**

| **Case** | **Type of synapse** | **Synapses on spine heads** | **Synapses on nf. spine heads** | **Synapses on spine necks** | **Synapses on aspiny dendritic shafts** | **Synapses on spiny dendritic shafts** | **Total synapses** |
| --- | --- | --- | --- | --- | --- | --- | --- |
| AB1 | AS | 45.3% (171) | 16.1% (61) | 1.1% (4) | 13.2% (50) | 24.3% (92) | 100% (378) |
|  | SS | 21.2% (7) | 6.1% (2) | 3.0% (1) | 18.2% (6) | 51.5% (17) | 100% (33) |
| AB3 | AS | 26.4% (110) | 18.7% (78) | 0.2% (1) | 25.7 (107) | 29.0% (121) | 100% (417) |
|  | SS | 14.3% (5) | 8.6% (3) | 5.7% (2) | 28.5% (15) | 42.9% (10) | 100% (35) |
| IF10 | AS | 42.3% (182) | 13.0% (56) | 0.9% (4) | 17.5% (75) | 26.3% (113) | 100% (430) |
|  | SS | 0.0% (0) | 0.0% (0) | 0.0% (0) | 23.1% (6) | 76.9% (20) | 100% (26) |
| M16 | AS | 45.5% (179) | 13.5% (53) | 0.8% (3) | 13.0% (51) | 27.2% (107) | 100% (393) |
|  | SS | 9.4% (3) | 3.1% (1) | 0.0% (0) | 18.8% (6) | 68.7% (22) | 100% (32) |
| IF1 | AS | 45.5% (130) | 11.5% (33) | 0.7% (2) | 24.8% (71) | 17.5% (50) | 100% (286) |
|  | SS | 2.5% (1) | 0.0% (0) | 0.0% (0) | 45.0% (18) | 52.5% (21) | 100% (40) |
| VK11 | AS | 38.4% (78) | 11.3% (23) | 1.5% (3) | 28.1 (57) | 20.7% (42) | 100% (203) |
|  | SS | 8.3% (1) | 0.0% (0) | 8.3% (1) | 25.0% (3) | 58.4% (7) | 100% (12) |
| VK16 | AS | 12.5% (4) | 31.3% (10) | 0.0% (0) | 53.1% (17) | 3.1% (1) | 100% (32) |
|  | SS | 0.0% (0) | 0.0% (0) | 0.0% (0) | 100% (2) | 0.0% (0) | 100% (2) |
| VK22 | AS | 44.0% (93) | 9.5% (20) | 0.5% (1) | 31.3% (66) | 14.7% (31) | 100% (211) |
|  | SS | 9.7% (3) | 3.2% (1) | 6.5% (2) | 35.4% (11) | 45.2% (14) | 100% (31) |
