# Supplementary figures and images for: 3D Analysis of the Synaptic Organization in the Entorhinal Cortex in Alzheimer’s Disease
Source: eNeuro. 2021 Jun 18;8(3):ENEURO.0504-20.2021. doi: 10.1523/ENEURO.0504-20.2021 (PMC8225407; doi:10.1523/ENEURO.0504-20.2021)

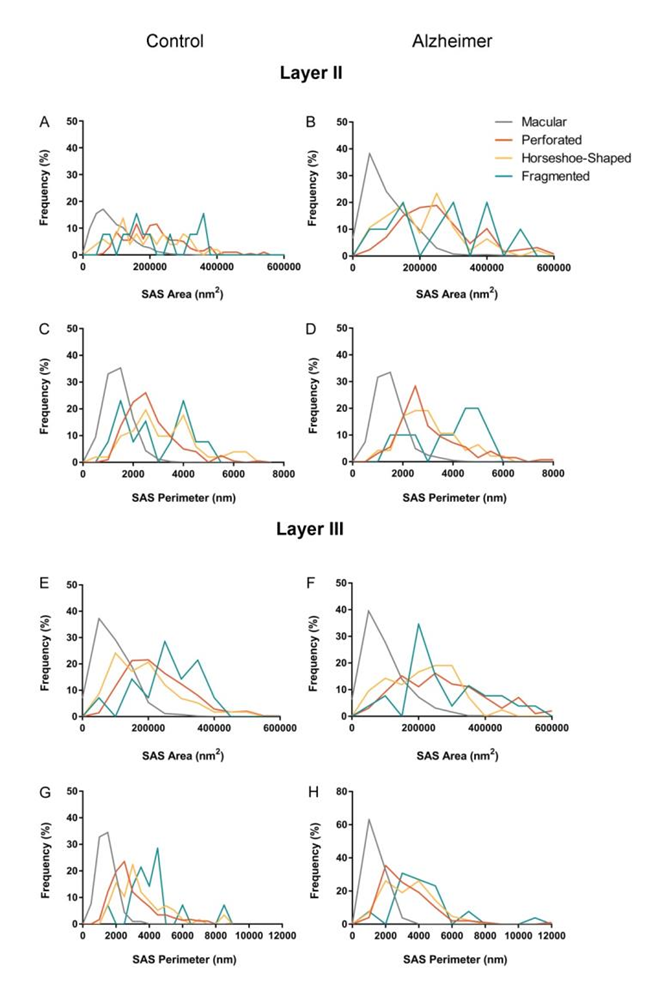

Supplement: Extended Data Figure 9-1 — Frequency histograms of the SAS area (A, B, E, F) and perimeter (C, D, G, H) of macular, perforated, horseshoe-shaped, and fragmented AS from control cases (A, C, E, G) and from AD cases (B, D, F, H), in Layers II and III of the EC. The SAS area and perimeter of macular synapses were significantly smaller than in perforated, horseshoe-shaped, and fragmented synapses (KW, p < 0.0001). No differences were found between control and AD cases (KS, p > 0.0001). Download Figure 9-1, TIF file. [file enu-eN-NWR-0504-20-s02.tif]

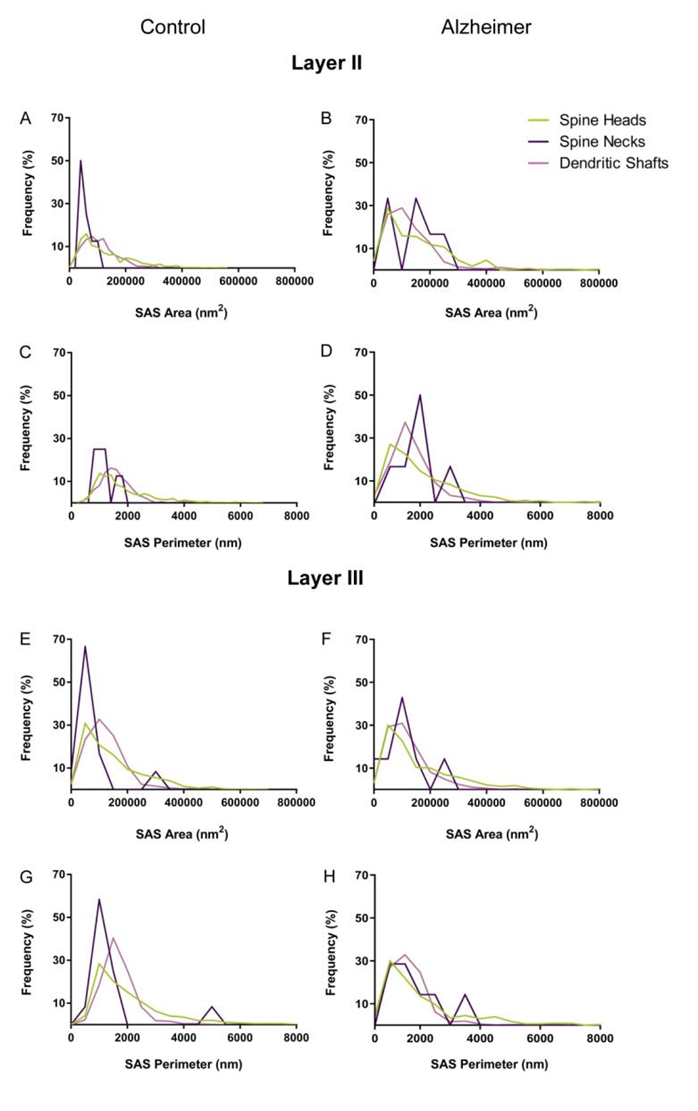

Supplement: Extended Data Figure 10-1 — Frequency histograms of the SAS area (A, B, E, F) and perimeter (C, D, G, H) of AS targeting spine heads, spine necks and dendritic shafts from control cases (A, C, E, G) and from AD cases (B, D, F, H), in both Layers II and III of the EC. No differences were found between groups (KS, p > 0.0001). Download Figure 10-1, TIF file. [file enu-eN-NWR-0504-20-s03.tif]
